# Supplementary material for: Revised phylogenetic analysis of the Aetosauria (Archosauria: Pseudosuchia); assessing the effects of incongruent morphological character sets
Source: PeerJ. 2016 Jan 21;4:e1583. doi: 10.7717/peerj.1583 (PMC4727975; doi:10.7717/peerj.1583)
Supplement: Supplemental Information 1 [file peerj-04-1583-s001.docx]

## APPENDIX A

### PHYLOGENETIC MATRIX:

MATRIX

'Postosuchus kirkpatricki' 0000000000000000000000000000000000000000000000000000-------------------00---------0

'Revueltosaurus callenderi' 00000010011001011010000110000000012001000110010110110000000000000-0000011---------2

'Adamanasuchus eisenhardtae' ???????????????????????????????????????????????????11101?????011010101?120?1-00000?

'Aetosaurus ferratus' 0110111001000211110102?101111100122?01000??000001?11110000000001010000112001-000002

'Apachesuchus heckerti' ???????????????????????????????????????????????????1310?0?00?0020100?0???????????2?

'Stagonolepis robertsoni' 111011101110110??0?100?00101211012200100001000001101110000000000010000112001-000002

'Stagonolepis olenkae' 11101120101??20??111000001111110122???00001??0011??11100??000000010000112001-00000?

'Calyptosuchus wellesi' ??????????????????????????0????????0000000100???0?0111010?000001010100112001-000002

'Scutarx deltatylus' ?????1????1????????101001???2??????0000000100???010111010?000001010102112001-000002

'Aetosauroides scagliai' ?0100110010??2????1100?1010100???1000110001001000001110000000000010000112001-000002

'Coahomasuchus kahleorum' ??????10???00??111?1??01000?0?0?120001000010?11????1110000000001010000112001-000002

'Desmatosuchus spurensis' ?1???121101110??0?1100101??????10??1020010000???10030000111111000012101123100211210

'Desmatosuchus smalli' 11221121101110010011??1012111101022002001000001110030000111111000012101123100211210

'Rioarribasuchus chamaensis' ????????????????????????????????????????????1??????210000?000012020000412102112012?

'Longosuchus meadei' ?1???121?0010????1?1011110111110122100000010010110010100111111000112101122001211210

'Lucasuchus hunti' ???????????????????????????????????????????????????11000111121000002103122000211?1?

'NCSM 21723' ???????????????????????????????????????????????????20100010?210?011?10?122000?11?1?

'Neoaetosauroides engaeus' 1110?12?0?1112???1?10100001121?1?22011000????01010?1100?00000000020100112001-000002

'Typothorax coccinarum' 011?012000?0???2?10100?10?01010102211001101000110011210201000012010000112101-020122

'Redondasuchus rineharti' ????????????????????????????????????120????????????121020?0000120100?01121?1-02012?

'Paratypothorax andressorum' ????????????????????????????????????????1011???????210120?000012120000212102112012?

'Paratypothorax sp.' ???????????????????????????????????????110111?0????210020?000012120000212102112012?

'Tecovasuchus chatterjeei' ???????????????????????10???????????0?0????0???????110120???0012120000112102112012?

'Sierritasuchus macalpini' ???????????????????????????????????0000010?0???????10100111111000012101122001211?1?

'SMNS 19003' 01101110010002121?1112???1??01?0?11????????????????2101?000000120200?0112102112012?

'Aetobarbakinoides brasiliensis' ???????????????????????????????????10?000000??01???11?0?0??000?001????1????????????

'Stenomyti huangae' 0111111000000201000111???10121?1122????????????????1110?0000?000020100112001-000001

'Polesinesuchus aurelioi' ???????????????????????0???????????00100??100000111111000?001000010?0011??????????2

;

ENDBLOCK;
