# Supplementary material for: Revised phylogenetic analysis of the Aetosauria (Archosauria: Pseudosuchia); assessing the effects of incongruent morphological character sets
Source: PeerJ. 2016 Jan 21;4:e1583. doi: 10.7717/peerj.1583 (PMC4727975; doi:10.7717/peerj.1583)
Supplement: Supplemental Information 2 [file peerj-04-1583-s002.docx]

**APPENDIX B**

### DESCRIPTION OF CHARACTERS

Many of these characters are taken from earlier phylogenetic studies by Parrish (1994), Heckert, Hunt & Lucas (1996), and Heckert & Lucas (1999), as well as an unpublished dissertation by Desojo (2005), although some have been modified to incorporate more recent understanding of aetosaurian anatomy. Thus, these are considered framework studies in addition to the phylogenetic work of Parker (2007), with each study building on the former as our knowledge of aetosaur anatomy and taxonomy has increased. Therefore, in this section rather than repeating all of the citations for every character, only the initial analysis where a character first appeared in its original form is listed.

***Institutional abbreviations*** – **AMNH**, American Museum of Natural History, New York, USA; **ANSP**, Academy of Natural Sciences of Drexel University, Philadelphia, Pennsylvania, USA; **CPE2**, Coleção Municipal, São Pedro do Sul, Brazil;  **DMNH**, Perot Museum of Natural History, Dallas, Texas, USA; **DMNH**, Denver Museum of Nature and Science, Denver, Colorado, USA; **FMNH**, Field Museum, Chicago, IL, USA; **FR**, Frick Collection, American Museum of Natural History, New York, USA; **MCCDP**, Mesalands Community College Dinosaur Museum, Tucumcari, New Mexico, USA; **MCSNB,** Museo Civico di Scienze Naturali Bergamo, Bergamo, Italy; **MCP**, Museo de Ciencias e Tecnología, Porto Alegre, Brazil**; MCZ**, Museum of Comparative Zoology, Harvard University, Cambridge, Massachusetts, USA; **MCZD**, Marischal College Zoology Department, University of Aberdeen, Aberdeen, Scotland, UK; **NCSM**, North Carolina State Museum, Raleigh, North Carolina, USA; **NHMUK**, The Natural History Museum, London, United Kingdom; **NMMNH**, New Mexico Museum of Natural History and Science, Albuquerque, New Mexico, USA; **MNA**, Museum of Northern Arizona, Flagstaff, Arizona, USA; **PEFO**, Petrified Forest National Park, Petrified Forest, Arizona, USA; **PFV**, Petrified Forest National Park Vertebrate Locality, Petrified Forest, Arizona, USA; **PVL**, Paleontología de Vertebrados, Instituto ‘Miguel Lillo’, San Miguel de Tucumán, Argentina; **PVSJ,** División de Paleontología de Vertebrados del Museo de Ciencias Naturales y Universidad Nacional de San Juan, San Juan, Argentina, **SMNS**, Staatliches Museum für Naturkunde, Stuttgart, Germany; **TMM**, Texas Memorial Museum, Austin, Texas, USA; **TTUP**, Museum of Texas Tech, Lubbock, Texas, USA; **UCMP**, University of California, Berkeley, California, USA; **ULBRA PVT**, Universidade Luterana do Brasil, Coleção de Paleovertebrados, Canoas, Rio Grande do Sul, Brazil; **UMMP**, University of Michigan, Ann Arbor, Michigan, USA; **USNM**, National Museum of Natural History, Smithsonian Institution, Washington, D.C., USA; **VPL**, Vertebrate Paleontology Lab, University of Texas at Austin, Austin, Texas, USA; **YPM**, Yale University, Peabody Museum of Natural History, New Haven, Connecticut, USA; **VRPH**, Sierra College, Rocklin, California, USA; **ZPAL,** Institute of Paleobiology of the Polish Academy of Sciences in Warsaw, Warsaw; Poland.

#### Cranial Characters

1. Premaxilla, anterior portion in dorsal view: tapers anteromedially (0); laterally expanded (1). Modified from Parrish, 1994: character 3 (in part). Figures 1a, c, d, f.

In aetosaurians such as *Stagonolepis robertsoni* (Walker, 1961) and *Desmatosuchus smalli* (TTU P-9024) the anterior end of the premaxilla is mediolaterally wide and maintains a nearly constant width until the apex, which is an inclined and mediolaterally expanded, described as a “shovel” by previous workers (e.g., Parrish, 1994). This expanded apex was considered to be present in all aetosaurs for which a premaxilla was preserved, therefore in earlier phylogenetic analyses the character was an autapomorphy of Aetosauria and parsimony uninformative (Parrish, 1994; Heckert & Lucas, 1999). Parker (2007) noted that an expanded apex was not present in *Aetosaurus* (SMNS 5770, S-16) and SMNS 19003. In these taxa the premaxillae gradually decrease in width anteriorly and bear flattened lateral margins (Small & Martz, 2013; Desojo & Schoch, 2014). The premaxillae of *Typothorax coccinarum* (PEFO 38001/YPM 58121), *Stenomyti huangae* (Small & Martz, 2013), and *Aetosauroides scagliai* (PVL 2073) also taper and lack the expanded apex.

2. Premaxilla, contact of posterior process with nasal: present, excludes maxilla from the margin of the external naris (0); absent, maxilla participates in the posterior margin of the external naris (1). Modified from Heckert & Lucas (1999), character 13. Figures 1a, g.

Contact between the nasal and premaxilla, excluding the maxilla from participation in the margin of the external naris is an apomorphy of Archosauriformes (Nesbitt, 2011). However, with the exception of *Aetosauroides scagliai* (PVL 2073), this contact is not present in aetosaurians and the maxilla forms a portion of the posterior and posteroventral borders of the external naris. In the referred skull of *Aetosauroides scagliai* (PVL 2073) the posterior process of the premaxilla underlies the entire length of the external naris contacting the ventral process of the nasal and excluding the maxilla from the border of naris (Casimiquela, 1961; Desojo & Ezcurra, 2011). Conversely, Heckert & Lucas (2002) considered figures by Casimiquela (1961, 1967) to be inconclusive (see below) and that the maxilla bounded a portion of the naris as in *Stagonolepis robertsoni* (Walker, 1961). Examination of PVL 2059 and PVL 2052 found that the maxilla is definitely excluded. Heckert & Lucas (1999) introduced this character and because they were unsure of the condition in *Aetosauroides scagliai*, scored it as unknown. Parker (2007) excluded this character without explanation; however, it is reintroduced here.

3. Premaxilla, tooth arrangement: teeth present along ventral surface of entire element (0); teeth present, but restricted to posterior half of the element (1); teeth absent (i.e., premaxilla edentulous) (2). Modified from Heckert, Hunt & Lucas (1996), character 21. Figures 1a, d, h.

Non-aetosaurian aetosauriforms such as *Revueltosaurus callenderi* have premaxillae with five alveoli present along the entire length of the element. At present, in all known aetosaurians with preserved premaxillae either the anterior portion (e.g., *Aetosaurus ferratus, Stagonolepis robertsoni*) or the entire element (*Desmatosuchus smalli*) lacks teeth. It was previously alleged that the premaxilla of *Typothorax coccinarum* is completely edentulous (e.g., Heckert, Hunt & Lucas, 1996) and was coded as such in all subsequent analyses (Heckert & Lucas, 1999; Parker, 2007; Desojo, Ezcurra & Kischlat, 2012); however, new specimens (e.g., PEFO 38001/YPM 58121) demonstrate that there are a minimum of four teeth present in the premaxilla of *Typothorax coccinarum*.

4. Premaxilla, tooth count (single ramus): 4 or more tooth positions (0); 3 tooth positions (1); edentulous (2). [Ordered] New character. Figures 1c, f.

In prior analyses all known aetosaurs either had edentulous premaxillae (e.g., *Desmatosuchus smalli*) or when teeth were present they numbered between 4 and 5 tooth positions (e.g., *Neoaetosauroides engaeus*, *Stagonolepis robertsoni*). However, the recently described *Stenomyti huangae* possesses only three premaxillary teeth (Small & Martz, 2013).

5. Premaxilla, dorsal surface of posterior process: smooth (0); with prominent dorsal tubercle that extends dorsally into the external naris (1). Small (2002). Figure 1a.

The presence of a tubercle on the dorsal surface of the premaxilla that projects into the naris of *Stagonolepis robertsoni* was briefly mentioned by Walker (1961); however, its possible phylogenetic significance was first recognized by Small (2002). In some taxa, such as *Stenomyti huangae*, the protuberance is weakly developed (Small & Martz, 2013); however it is still scored as present for this study.

6. External naris, anteroposterior length: less than the anteroposterior length of the antorbital fenestra (0); length is greater than or equal to that of the antorbital fenestra (1). Heckert & Lucas (1999), character 8. Figures 1g, h.

In *Postosuchus kirkpatricki* the anteroposterior length of the external naris is less than that of the antorbital fenestra (Weinbaum, 2011); however, it is longer in all known aetosaurians so presently this character is parsimony uninformative within Aetosauria as the extreme length found in aetosaurians is an autapomorphy of that group (Parker, 2007).

7. Maxilla, lateral surface, longitudinal ridge: present, rounded and bulbous (0); present, sharp (1); absent, lateral surface is smooth (2). Modified from Nesbitt (2011), character 26. Figures 1d; 2a.

Character state 2 refers to specimens with a smooth lateral surface of the maxilla ventral to the antorbital fenestra, as well as specimens that possess an antorbital fossa where the fossa rim is not raised above the surface of the maxilla (Nesbitt, 2011). Specimens with a fossa rim that is raised above the surface of the maxilla are scored as 1. A third character state where the raised ridge was bulbous (0) is only found in the outgroup taxon *Postosuchus kirkpatricki* (Nesbitt, 2011).

8. Maxilla, ventral portion of antorbital fossa in lateral view: dorsoventrally deep, more than 1/3 of the total element height (0); dorsoventrally shallow, less than 1/3 of the total element height, or absent (1). New character. Figures 1a; 2a.

Some aetosaurians (e.g., *Stagonolepis robertsoni*) possess a dorsoventrally deep antorbital fossa ventral to the antorbital fenestra; whereas in others (e.g., *Desmatosuchus spurensis*) the ventral portion of the fossa is extremely shallow.

9. Nasal, shape of anterior margin in dorsal view: tapering (0); maintains an equal width (1).New character. Figures 1b, e; 2b.

In some aetosaurians (e.g., *Stenomyti huangae*) the nasals reduce in transverse width anteriorly, tapering to a point dorsal to the premaxilla (Small & Martz, 2013). In others such as *Desmatosuchus smalli* (TTU P-9024), the nasals maintain a nearly constant width along the entire anterior portion, contacting the posterior margin of the premaxillae.

10. Nasal, lateral margin: does not form part of the dorsal border of the antorbital fossa (0); forms part of the dorsal border of the antorbital fossa (1). Nesbitt (2011), character 37. Figures 1a, g; 2a.

In taxa without an extensive antorbital fossa, the ascending process of the maxilla and the anterior portion of the lacrimal meet to exclude the nasal from bordering the antorbital fossa (Nesbitt, 2011).

11. Nasals, posterior portion of the midline suture area: triangular depression (formed at the midline) (0); flat or convex (1). New character. Figures 1b, e; 2b.

In *Stenomyti huangae* the lateral margins of the nasals are raised and this raised area widens anteriorly, causing a triangular depression to form on the posteromedial portion of the nasals (Small and Martz, 2011: fig. 11c). This triangular depression is also apparent in *Aetosaurus ferratus* (Schoch, 2007: fig. 8c), *Aetosauroides scagliai* (PVL 2059), and *Longosuchus meadei* (TMM 31185-97). In contrast, the nasals of *Stagonolepis olenkae* (AbIII/2000) lacks this depression, as do the nasals of *Desmatosuchus* (e.g., UMMP 7476).

12. Jugal, lateral view: ventral margin is nearly horizontal (0); ventral margin is strongly posteroventrally inclined (1). Modified from Nesbitt (2011), character 74, and Heckert & Lucas (1999), character 14. Figures 1a, d.

In lateral view the ventral margin of the jugal is oriented nearly horizontally in most archosauriforms (Nesbitt, 2011), including aetosaurians such as *Aetosaurus ferratus* (Schoch, 2007) and *Stenomyti huangae* (Small & Martz, 2013). In other aetosaurians such as *Desmatosuchus spurensis* (UMMP 7476) and *Longosuchus meadei* (TMM 31185- 97) the jugal is strongly inclined anterodorsally so that the quadrate condyle is situated ventrally to the maxillary tooth row. *Stagonolepis olenkae* has a nearly horizontal ventral margin of the jugal; however, the jugal and quadratojugal are unknown for that taxon and were reconstructed using bones from *Neoaetosauroides* and *Desmatosuchus* (Sulej, 2010:867, 869). Likewise the jugals are missing in the skull of *Aetosauroides scagliai* (PVL 2059) and *Scutarx deltatylus* (PEFO 34616). A referred specimen of *Aetosauroides scagliai* (PVL 2052) preserves the anterior portion of the jugal; however, not enough is preserved to determine if it was inclined or nearly horizontal.

13. Jugal, anterior process: excluded from the border of the antorbital fenestra by contact between the lacrimal and maxilla (0); contributes to the border of the antorbital fenestra. (1). New character. Figures 1a, d.

In *Stenomyti huangae* (Small & Martz, 2013) the posterior portions of the lacrimal and maxilla contact each other, excluding the anterior portion of the jugal from contributing to the border of the antorbital fossa. In *Desmatosuchus smalli* (Small, 2002) the posterior portions of the lacrimal and maxilla are separated from each other by the forward projection of the jugal into the margin of the antorbital fenestra.

14. Postfrontal, contact with parietal: absent (0); restricted by a posterolateral process of the frontal (1); extensive (2). [Ordered]. Desojo (2005), character 12. Figures 1b, e.

In some taxa (e.g., *Aetosaurus ferratus, Stenomyti huangae*), the postfrontal and parietal share an extensive border along the anterolateral margin of the parietal (Schoch, 2007; Small & Martz, 2013). In *Stagonolepis robertsoni*, this shared border is greatly restricted by a posterolateral process of the frontal that nearly contacts the anterodorsal corner of the dorsal process of the postorbital (Walker, 1961). In *Desmatosuchus smalli*, the postfrontal and parietal are separated from each other by a strong contact between the postorbital and the frontal (Small, 2002).

15. Postorbital: contact with quadratojugal - absent (0); present (1). Nesbitt (2011), character 64. Figures 1d, h.

In the majority of aetosaurians the postorbital and quadratojugal are separated from each other by an anterior process of the squamosal. However, in SMNS 19003 the squamosal process is reduced and there is extensive contact between the postorbital and quadratojugal. Schoch (2007) noted contact between the two bones in some specimens of *Aetosaurus ferratus*, and following Nesbitt (2011), *Aetosaurus ferratus* is coded as possessing character state 1.

16. Quadratojugal, anterior process: absent (0); forms ventral margin of lateral temporal fenestra (1); underlies jugal and is excluded from the lateral temporal fenestra (2). New character. Figure 1h.

In most aetosaurians, an anterior projection of the quadratojugal separates the dorsal portion of the posterior process of the jugal from the lateral temporal fenestra. However, in SMNS 19003, the posterior process of the jugal overlies the anterior process of the quadratojugal forming the entire fenestra border. *Neoaetosauroides* has a similar condition (state 1: Desojo & Báez, 2007); however, of the two referred skulls, one has had the lateral temporal fenestra artificially enlarged and the other is actually impressions of the bones in soft tissue, thus it is difficult to tell the actual condition and it is scored as unknown. In *Stagonolepis robertsoni* the anterior projection of the quadratojugal forms the ventral margin of the lateral temporal fenestra; however, this reconstruction is based on *Aetosauroides ferratus* (Walker, 1961: 127). The jugal and quadratojugal are unknown for *Stagonolepis olenkae* (Sulej, 2010).

17. Quadratojugal, anterior margin: lacks dorsal anteroprocess between the posterior process of the jugal and the lateral temporal fenestra (0); distinct anterior facing notch in the middle of the anterior margin for reception of posterior process of the jugal (1). New character. Figure 1a, h.

In *Revueltosaurus callenderi* (PEFO 34561) and some aetosaurians such as *Coahomasuchus kahleorum* (TMM 31100-437) the anterior margin of the quadratojugal bears a distinct anteriorly opening notch for reception of a posterior process of the jugal. Other aetosaurs such as *Stenomyti huangae* lack this notch and the posterior process of the jugal underlies the quadratojugal (Small & Martz, 2013).

18. Quadrate foramen, position: between the quadrate and the quadratojugal (0); completely within the quadrate (1). Modified from Nesbitt (2011), character 79. Figure 1a.

In *Stagonolepis robertsoni* the quadrate foramen is situated between the quadratojugal and the quadrate (Walker, 1961: 122). In *Coahomasuchus kahleorum* (TMM 31100-437) the foramen is entirely within the quadrate body.

19. Parietals/ frontals - transverse width at anteroposterior mid-points: parietal wider (0); frontal wider (1). New character. Figures 1b, e; 2b.

In *Aetosaurus ferratus* (Schoch, 2007) and *Stenomyti huangae* (Small & Martz, 2013) the transverse width of the parietals at their midpoint is greater than that of the frontals at their midpoint. In *Desmatosuchus smalli* (Small, 2002) and *Stagonolepis robertsoni* (Walker, 1961) the frontals are wider than the parietals at mid-point.

20. Supratemporal fenestra, position: only exposed in dorsal view (0); dorsolaterally or laterally oriented and visible in lateral view (1). Modified from Heckert & Lucas (1999), character 10. Figures 1a, h.

In *Revueltosaurus callenderi* (PEFO 34561) the supratemporal fenestrae are exposed dorsally which is the typical archosauriform condition; however, in aetosaurians the squamosal and the postorbital-squamosal bar have shifted ventrally and the supratemporal fenestra is broadly exposed in lateral view. All aetosaur taxa that can be scored for this character have laterally exposed supratemporal fenestra so presently the character offers no in-group resolution (Parker, 2007). The supratemporal fenestra is completely exposed laterally in *Aetosauroides scagliai* (PVL 2073), a potential autapomorphy of that taxon.

21. Supratemporal fenestra, shape in lateral view: horizontal orientation of parietal forms a round or oval fenestra (0); strong posteroventral orientation of the posterior portion of the parietal forms a triangular fenestra (1). New character. Figures 1d, g.

The shape of the supratemporal fenestra is variable within Aetosauria and formed by the orientation of the parietal. *Desmatosuchus spurensis* (UMMP 7476) has a round fenestra, whereas it is triangular in SMNS 19003. In *Neoaetosauroides engaeus* (PVL 5698) the fenestra is strongly oval.

22. Supratemporal fenestra, anteroposterior diameter: larger than or nearly the same diameter as orbit (0); roughly half the size of the orbit (1); less than half the diameter of the orbit (2). [Ordered]. New character. Figures 1a, d, g.

The size of the supratemporal fenestra is variable in aetosaurs. It is nearly the size of the orbit in *Desmatosuchus spurensis* (UMMP 7476), roughly half the size of the orbit in *Neoaetosauroides engaeus* (PVL 5698), and much smaller than the orbit in SMNS 19003.

23. Post-temporal fenestra: present (0); absent (1). New character. Figures 1b, e.

In aetosaurs the posttemporal fenestra is a broad slit-like opening in the back of the skull between the ventral portion of the posteroventrally sloping flange (*sensu* Walker, 1961:114) of the parietal and the dorsal margin of the paroccipital process of the opisthotic. This fenestra is present in *Stagonolepis olenkae* (Sulej, 2010) and *Scutarx deltatylus* (PEFO 34616). Conversely, the fenestra is not present in *Desmatosuchus spurensis* (Case, 1922). This character is difficult to score in articulated specimens where the nuchal and anterior cervical paramedian osteoderms cover the back of the skull (e.g., *Aetosaurus ferratus*).

24. Basicranium, basal tubera: nearly or completely connected medially (0); clearly separate (1). Modified from Nesbitt (2011), character 104. Figures 3a-c.

In *Aetosauroides scagliai* (PVSJ 326) the basal tubera are distinctly separated by a broad anteroposterior trough between the occipital condyle neck and the basisphenoid recess. In *Scutarx deltatylus* (PEFO 34616) the basal tubera contact each other along the midline. In *Desmatosuchus spurensis* (UMMP 7476) are situated very closely together and are connected by a medial ridge. This latter condition is scored the same as a midline contact.

25. Basioccipital, distance between basal tubera and basipterygoid processes: widely separated anteroposteriorly (0); closely situated or nearly touching (1). Similar to Nesbitt (2011), character 103. Figures 3a-c.

In *Aetosauroides scagliai* (PVSJ 326) and *Stagonolepis robertsoni* (MCZD 2) the basal tubera and basipterygoid processes are widely separated anteroposteriorly the result of elongation of the parabasisphenoid. In *Desmatosuchus spurensis* (UMMP 7476) and *Scutarx deltatylus* (PEFO 34616), the basal tubera and basipterygoid processes are very closely situated anteroposteriorly, and this results in a foreshortened parabasisphenoid. This distance is the best way to quantify the differences in relative length of the parabasisphenoid between aetosaurians.

26. Dentary, dorsal and ventral posterior processes in lateral view: roughly equal lengths (0); upper process more elongate (1); lower process more elongate (2). New character. Figures 1a, d, h.

In most aetosaurians the posterior portion of the dentary splits into two posterior processes that are situated dorsal and ventral to the lateral mandibular fenestra. In lateral view these two processes are of roughly equal length in *Neoaetosauroides engaeus* (PVL 3525); however, in *Stagonolepis olenkae* (ZPAL AbIII/573) the dorsal process is much more elongate than the lower process. The reverse is found in *Desmatosuchus smalli*, where the ventral process in longer than the dorsal process (Small, 2002). The dentary of *Coahomasuchus kahleorum* (TMM 31100-437) also bears a median posterior (third) process as in *Revueltosaurus callenderi* (PEFO 34561), but for this character they are both scored as 0 as all of the processes are of equal length.

27. Dentary, tooth count: 9 or more (0); fewer than 9 (1). Parker (2007), character 9; modified from Heckert & Lucas (1999), character 16. Figures 1a, g.

Dentary tooth counts are variable across Aetosauria; however, the range of alveoli present seems to border on nine, with *Desmatosuchus smalli*, *Neoaetosauroides engaeus*, *Aetosaurus ferratus*, and *Longosuchus meadei* possessing between six and eight dentary tooth positions and *Aetosauroides scagliai* and *Stagonolepis robertsoni* having nine or ten. The original character of Heckert & Lucas (1999) used 10 positions as the division between character states, but Parker (2007) changed it to nine without discussion. The use of nine teeth is retained here as *Stagonolepis robertsoni* has nine or 10 teeth (Walker, 1961). Furthermore, *Stenomyti huangae* has a minimum of nine positions in an incomplete lower jaw (Small & Martz, 2013) so the choice of nine as the division simplifies these character state codings. There are seven to nine positions in *Stagonolepis olenkae* (Sulej, 2010), but the average value is used and scored as state 1. Parker (2007) followed Heckert & Lucas (1999) scored *Aetosaurus ferratus* as state 0; but despite that these teeth are poorly exposed in all known specimens, *A. ferratus* apparently has only seven or eight dentary tooth positions (Walker, 1961; Schoch, 2007). *Typothorax coccinarum* has more than nine dentary teeth based on PEFO 38001/YPM 58121. *Coahomasuchus* *kahleorum* can also be scored as 0 based on specimen TMM 31100-437.

28. Dentary, anterior half of dorsal margin: with teeth/alveoli (0); edentulous (1). Parrish (1994), character 9. Figures 1a, g, h.

In all aetosaurians with preserved dentaries, the anterior portions are edentulous, so this character is presently parsimony uninformative with respect to in-group relationships (Parker, 2007). However, *Revueltosaurus callenderi*, which is the sister taxon of Aetosauria (Nesbitt, 2011), bears alveoli for the entire length of the dentary so the possibility exists for an aetosaurian to possess the plesiomorphic character state. Earlier analyses (Parrish, 1994; Heckert, Hunt & Lucas, 1996; Heckert & Lucas, 1999) scored *Aetosaurus ferratus* as having teeth in the anterior portion of the dentary, but Walker (1961) noted that the anterior portion of the dentary was edentulous. This was also noted by Schoch (2007).

29. Mandibular ramus, ventral margin in lateral view: The ventral margin of the ramus is gradually convex (0); a 'chin' is present, formed by a ventral inflexion of the splenial, which is exposed ventral to the dentary (1); a 'chin' is present, formed by a ventral inflexion of the dentary, which covers the splenial (2). New character. Descriptive terminology after Desojo & Ezcurra (2011). Figures 1a, d, g, h.

A ‘classic’ character of aetosaurians is the ventral inflexion of the mandibular ramus in some taxa forming a prominent ‘chin’, which is part of Walker’s (1961) description of the element as ‘slipper-shaped’ (see discussion below). When present, this ‘chin’ is usually formed by the ventral inflexion of the splenial, extending below the concave ventral margin of the dentary. In this arrangement the ventral portion of the splenial is visible in lateral view. *Stenomyti huangae* possesses a different arrangement, where the ventral inflexion is actually on the dentary and the splenial is not visible in lateral view (Small & Martz, 2013). This may also be the case in *Neoaetosauroides engaeus* (Small & Martz, 2013). Examination of PVL 3525, a right mandibular ramus, shows that the anterior portion is mostly reconstructed in plaster, but there is a slight ventral inflexion of the dentary where the bone ends. A referred specimen of *Neoaetosauroides engaeus* (PULR 108) also shows that the ventral margin of the dentary bears the ventral inflexion (Desojo & Báez, 2007; Desojo & Vizcaíno, 2009).

30. Dentary, anterior end in lateral view: rounded (0); tapers to an acute point (1). Modified from Heckert & Lucas (1999a), character 15. Figures 1a, d, g-h.

Walker (1961) was the first to describe the acute termination of the anterior portion of the dentary as contributing to the ‘slipper-shape’ of the mandibular ramus. The presence or absence of this shape was used as a phylogenetic character by Heckert & Lucas (1999) and Parker (2007), although all aetosaurians with preserved dentaries were scored as possessing that character so it was parsimony uninformative. The condition ‘slipper-shape’ is separated into two distinct characters (also see Character 29). Desojo & Ezcurra (2011) noted that the dentary of *Aetosauroides scagliai* was slender, lacking the ventral inflexion found *Stagonolepis robertsoni* (Walker, 1961), and therefore was not ‘slipper-shaped.’ Furthermore, although the anterior end of the dentary of *Aetosaurus scagliai* (PVL 2059) is distorted, it clearly was not acute and accordingly should be scored as bearing the 0 state (Desojo, Ezcurra & Kischlat, 2012).

31. Surangular, dorsal margin: smooth (0); prominent rounded tuber (1). New character. Figure 4.

The surangular of *Stagonolepis olenkae* (ZPAL AbIII/578/34) bears a distinct rounded tuber on the dorsal surface dorsal to the lateral mandibular fenestra. A similar tuber is also present *in Stagonolepis robertsoni*, which Walker (1961) attributed to muscle or tendon attachment, most probably the *musculus adductor mandibulae externus* (Desojo & Vizcaíno, 2007). This tuber is absent in other specimens such as *Aetosaurus ferratus* (Schoch, 2007).

32. Articular, retroarticular process: height is greater than or equal to the length (0); longer than high (1). Desojo (2005), character 11. Figures 1a, 4.

In *Longosuchus meadei* (TMM 31185-84B) the retroarticular process of the articular is anteroposteriorly short and dorsoventrally tall. In contrast, the retroarticular process of *Desmatosuchus spurensis* (MNA V9300) is more elongate so that its anteroposterior length is greater than the dorsoventral height (Parker, 2008).

33. Articular, dorsolateral surface: smooth (0); dorsally projecting tuber (1). New character. Figures 1a, d; 4.

The articular of *Stagonolepis olenkae* bears a pronounced dorsally projecting tuber (=articular projection) that is readily visible in lateral and medial views (Sulej, 2010:figs. 6, 7). It is also present in *Coahomasuchus kahleorum* (TMM 31100-437) and *Longosuchus meadei* (TMM 31185-98). This tuber is absent in *Desmatosuchus spurensis* (MNA V9300) where the dorsal surface of the articular is smooth. There is a mound in *Desmatosuchus smalli* (TTU P-9023) but not the sharp, well-developed tuber found in other taxa.

34. Tooth, maxillary, root and crown base shape in occlusal view: narrow, mediolaterally compressed (0); oval, but not strongly mediolaterally compressed (1); conical (2). [Ordered] Modified from Parrish (1994), character 8. Figure 5.

The teeth of *Postosuchus kirkpatricki* (e.g., TTU P-9000) are mediolaterally compressed in occlusal view. No aetosaurian possesses teeth that are as medially compressed. The teeth of SMNS 19003 are oval in occlusal view, but not mediolaterally compressed, whereas in other aetosaurians the teeth are conical or round in occlusal view (e.g., *Desmatosuchus smalli*, TTU P-9023).

35. Tooth, maxillary, crown shape in labial/lingular view: fully recurved, anterior edge is convex and posterior edge is straight or concave (0); bulbous and partly recurved, anterior edge is concave, posterior edge straight (1); bulbous with pointed or slightly recurved tips (2). Figures 5a-d.

Aetosaurian teeth are quite variable in their anatomy, even within an individual skull, and this variability is difficult to capture as discrete states for a phylogenetic character. The first study to try to capture this was Parrish (1994), who divided aetosaurian tooth form into two states, recurved or conical. Heckert & Lucas (1999) retained Parrish’s original character and added a second character: teeth unreduced, mediolaterally compressed, or reduced in size, nearly conical. However, this essentially was a duplication of Parrish’s character and indeed all taxa coded for these characters were coded similarly with the exception of *Aetosaurus ferratus*.

In each of these analyses *Aetosaurus ferratus* was coded as possessing recurved teeth (Parrish, 1994) and recurved conical teeth (Heckert & Lucas, 1999). The only other taxon coded for recurved, mediolaterally compressed teeth in the Heckert & Lucas (1999) analysis was the rauisuchid *Postosuchus kirkpatricki.* However, Walker (1961) had noted earlier that the teeth of *Aetosaurus ferratus* were actually conical, with a bulbous base of the crown, and that only the apices were recurved. Parker (2007) tried to capture this variation with three character states; 1) mediolaterally compressed and recurved; 2) bulbous and conical with recurved tips; and 3) bulbous and conical lacking recurved tips, but this actually describes two non-homologous characters.

Since that time Schoch (2007) published a full description of *Aetosaurus ferratus*, including the dentition. He noted that none of the teeth of *Aetosaurus ferratus* were recurved and that they were conical and more similar to the teeth of other aetosaurs, but they all had a well-curved anterior edge unlike other aetosaurians except for *Stenomyti huangae* and SMNS 19003. However, both *Aetosauroides scagliai* and the “carnivorous aetosaur” (=*Coahomasuchus kahleorum*) of Murry & Long (1996) have conical teeth that are fully recurved unlike the mediolaterally compressed, recurved teeth of *Postosuchus kirkpatricki*. Thus, these states are divided between two distinct characters, one (character 33) describing the tooth shape in occlusal view, and the other (character 34) describing crown shape in labio-lingular view.

#### Postcranial Characters

36. Cervical centra, ventral surface, at midline: keeled (0); smooth (1). Heckert & Lucas (1999), character 20. Figure 6b, c, f.

Many archosauriforms have prominent anteroposteriorly sharp flanges of bone (keels) on the ventral surface of the cervical centra (Nesbitt, 2011). However, some aetosaurs (e.g*., Desmatosuchus spurensis*) have smooth ventral surfaces (Parker, 2008).

37. Cervical vertebrae, length: anteroposteriorly shorter (more than 50%) than dorsoventrally tall (0); anteroposteriorly shorter (less than 50%) than dorsoventrally tall (1). New character. Figures 6b, e.

Aetosaurians differ from many archosauriforms in possessing cervical centra that are much shorter anteroposteriorly, than they are tall. However, some aetosaurians have cervical centra that are even anteroposteriorly shorter such as *Typothorax coccinarum* (Long and Murry, 1995).

38. Cervical vertebrae, centrum, shape of articular face: transversely oval (0); circular (1); subrectangular (2). Desojo (2005), character 17. Figures 6a, d.

The shape of the anterior articular face of the cervical centrum is variable in aetosaurians, circular in *Aetobarbakinoides brasiliensis* (CPE2 168), *Sierritasuchus macalpini* (UMMP V60817), and *Stagonolepis robertsoni* (Walker, 1961); a shallow, wide oval in *Neoaetosauroides engaeus* (PVL 3525) and *Calyptosuchus wellesi* (UCMP 27225); and sub-rectangular in *Desmatosuchus spurensis* (MNA V9300; Desojo, Ezcurra & Kischlat, 2012).

39. Cervical and trunk vertebrae, lateral surfaces of centra: concave or flat (0); concave with deep fossae (1). Desojo & Ezcurra, 2011. Figures 6e; 7d.

The cervical and trunk vertebrae of *Aetosauroides scagliai* bear prominent lateral fossae that cover much of the lateral surface of the centrum (Desojo & Ezcurra, 2011).

40. Trunk vertebrae, transverse processes: short, less than twice as wide as the centrum (0); elongate, more than twice as wide as the centrum (1). Heckert & Lucas (1999), character 18. Figures 7b, c, f.

The mid-trunk vertebrae of *Paratypothorax* sp. (TTU P-9169) and *Typothorax coccinarum* (e.g., TTU P-9214) have elongate transverse processes. The transverse process width is 2.5 times the centrum width in TTU P-9214 (Martz, 2002). These elongate transverse processes appear to coincide with the more discoidal carapace in *T. coccinarum* and *Paratypothorax*. Long & Murry (1995) considered the transverse processes of *Calyptosuchus wellesi* to be extremely long; however, they were examining the posterior dorsal vertebrae, which have ribs fused to the transverse processes and *Calyptosuchus wellesi* should be coded as having short transverse processes because the mid-dorsals less than twice the width of the centrum (Parker, 2007). In the specimen of *Calyptosuchus wellesi* with the longest transverse processes (UMMP 7470) the process is about 1.9 times the width of the centrum.

41. Mid-trunk vertebrae, neural spine height (from the base of the spine): greater than the height of the centrum (to the neurocentral suture) (0); equal to or less than the height of the centrum (1). Heckert & Lucas (1999), character 19. Figures 7b, c, f.

In *Desmatosuchus* (e.g., MNA V9300) and *Typothorax coccinarum* (e.g., TTU P-9124) the heights of the neural spines of the presacral vertebrae are less than the height of the centrum. In contrast, *Stagonolepis robertsoni* (Walker, 1961) and *Neoaetosauroides engaeus* (Desojo & Báez, 2005) have tall neural spines that are more than the height of the centrum. This character is restricted to the mid-trunk series because in *Scutarx deltatylus*, the anterior and mid-trunk vertebrae have neural spines that are shorter than the centrum. However, around the position of the 13^th^ trunk vertebra the neural spine and centrum heights transition to be roughly equal and in the more posterior vertebrae the neural spine becomes taller than the centrum. This variation does not occur in other specimens with short neural spines such as *Desmatosuchus spurensis*, as the ratio remains constant through the entire vertebral column (Parker, 2008).

42. Trunk vertebrae, zygadiapophyseal laminae, connecting the diapophysis to the zygapophyses: present (0); absent (1). Desojo (2005), character 19. Figures 7a, b, d.

The cervical and trunk vertebrae of some aetosaurians (e.g., *Desmatosuchus spurensis*, MNA V9300) bear distinct vertebral laminae (Parker, 2008). Four sets are present in the cervical vertebrae of *Desmatosuchus spurensis*, following the terminology of Wilson (1999) they are the 1) the acdl, anterior centrodiapophyseal lamina, which originates on the diapophysis and terminates on the anterior margin of the neurocentral suture; 2) the pcdl, posterior centrodiapophyseal lamina, which originates on the diapophysis and terminates on the posterior margin of the neurocentral suture; 3) the podl, the postzygadiapophyseal lamina, which originates on the diapophysis and terminates on the postzygapophysis; and the 4) prezygadiapophyseal lamina, which originates on the diapophysis and terminates at the prezygapophyses. Furthermore, the trunk vertebrae of *Desmatosuchus spurensis* possess an additional two spinozygapophyseal laminae (*sensu* Wilson, 1999); 1) the spol, spinopostzygapophyseal lamina, which originates on the postzygapophysis and terminates on the posterior face of the neural spine; and 2) the sprl, the spinoprezygapophyseal lamina, which originates on the prezygapophysis and terminates on the anterior face of the neural spine. The presacral vertebrae of *Typothorax coccinarum* (TTU P-9214) and *Paratypothorax andressorum* (NHMUK 38070) possess all four laminae and the zygadiapophyseal laminae are extremely robust and confluent with the pre- and postzygapophyses.

A pair of laminae previously unrecognized in aetosaurs is present in the cervical vertebrae of *Scutarx deltatylus*. These laminae originate on the posteroventral surface of the postzygapophyses and form two sharp ridges that meet at the dorsomedial margin of the neural canal. These appear to be homologous to the intrapostzygapophyseal laminae (tpol) of Wilson (1999) found in saurischian dinosaurs.

Aetosaurian vertebrae generally tend to be poorly preserved, crushed and broken, or often are covered by the carapace and inaccessible. Furthermore, delicate portions of the bone such as accessory processes and laminae are often broken away making determination of their presence/absence difficult. Nonetheless, all known aetosaurians have presacral vertebrae with zygadiapophyseal laminae (except possibly *Polesinesuchus aurelioi*, Roberto-da-Silva et al., 2014) suggesting the presence may be an aetosaurian apomorphy. Future versions of this character may focus on the presence or absence of specific laminae (e.g., intrapostzygapophyseal laminae) once the presence or absence of general laminae has been demonstrated for most aetosaurians.

43. Trunk vertebrae, well-developed intervertebral articulations (hyposphene/hypantrum): present (0); absent (1). Figure 7f.

*Desmatosuchus spurensis* (e.g., MNA V9300) possesses a well-developed hyposphene and hypantrum in the trunk vertebrae (Parker, 2008). Presently the only other known taxon with well-developed processes is *Aetobarbakinoides brasiliensis* (Desojo, Ezcurra & Kischlat, 2012). TTU-P09416, a posterior presacral vertebra of *Paratypothorax* sp., has a very slight posterior projection at the base of the postzygapophyses (Figure 8b) that may represent an incipient hyposphene as it corresponds to a slight indentation in the ventral bar between the prezygapophyses, but this is not the same as the extremely well-developed processes in *Desmatosuchus* and *Aetobarbakinoides* and therefore is scored as 0. However if this feature is found to be present in more aetosaurian specimens, a new state could be added for this character.

44. Posterior trunk vertebrae (positions 14-16), ventral surface: smooth, rounded (0); lateral faces meet to form sharp edge or keel (1). New character. Figure 8a.

In the posterior trunk vertebrae of *Paratypothorax* sp., the lateral surfaces of the centrum meet to form a sharp ventral keel on the ventral surface of the centrum. This is best demonstrated by TTU P-09416, a posterior dorsal vertebra from the Post Quarry of Texas (Figure 8a). A ventral keel is present but weakly formed in NHMUK 38070, a posterior trunk vertebra of *Paratypothorax andressorum*.

45. Anterior caudal vertebrae (positions 1-12), origin point of caudal ribs: at the level of neural arch (0); near the base of the centrum (1). New character. Figure 7e, g.

In most aetosaurians the caudal ribs originate from the neural arch (e.g., *Desmatosuchus spurensis*, MNA V9300), but in *Paratypothorax* sp. (PEFO 3004) the caudal ribs originate low down on the centrum. An isolated caudal vertebra (GR 174) from the Hayden Quarry at Ghost Ranch New Mexico possesses caudal ribs situated low on the centrum and is most likely referable to *Rioarribasuchus chamaensis*, which occurs in the quarry (Irmis et al., 2007).

46. Coracoid, posterolateral thickening below glenoid lip that divides coracoid into posterior and lateral faces ('subglenoid pillar'): present (0); absent (1). Desojo (2005), character 20. Figures 9a-b.

Walker (1961:145) described the subglenoid region of the coracoid of *Stagonolepis robertsoni* as bearing “a depressed area bounded in front by a stout pillar which makes a slight projection in the medial margin and thus divides the outer surface into two areas”. The first area contains the coracoid foramen and receives the insertion of *M. supracoracoideus*; whereas the second area is the insertion area for *M. coracobranchialis* (Desojo, 2005). A subglenoid pillar is also present in *Typothorax coccinarum* (Long & Murry, 1995) and absent in *Aetosauroides scagliai* and *Longosuchus meadei* (Desojo, 2005). A nearly complete scapulocoracoid of *Coahomasuchus kahleorum* (TMM 31100-437) lacks a prominent subglenoid pillar.

47. Humerus, distal end, lateral side of the ectepicondyle: proximodistally oriented groove present (0); proximodistally oriented foramen present (1). New character, but similar to Nesbitt (2011), character 234. Figures 9c-e.

The ectepicondylar foramen is present at the distal end of the humerus where it serves as a passage for the median (ulnar) nerve and the brachial artery (Landry, 1958). In stem- amniotes the opening is a foramen completely enclosed by bone in mature individuals (Romer, 1956). However; in some aetosaurs (e.g., *Aetosaurus ferratus*), the foramen is open laterally and thus a groove instead of a true foramen. A laterally open ectepicondylar groove is also found in *Revueltosaurus callenderi* (PEFO 34561), suggesting that this is the plesiomorphic state for aetosaurians. Nesbitt (2011) notes that a groove is also present in phytosaurs and some paracrocodylomorphs.

48. Humerus, proximal head: expanded medially, but lacks significant lateral expansion (0); broadly expanded transversely, with significant lateral expansion (1). New character. Figures 9d-e.

The proximal head of the humerus of *Aetosauroides scagliai* (PVL 2073) is moderately expanded (about one-third the element length), with almost all of the expansion medially, and almost no lateral expansion. In contrast, the humeral head of *Desmatosuchus smalli* (TTU P-9024) is nearly one-half the element length. Not only is the head expanded medially, but there is also a significant lateral expansion.

49. Ilium, orientation of acetabulum: opens fully or mostly ventrally (0); opens fully or mostly laterally (1). Modified from Desojo (2005), character 23. Figures 10a, c.

As noted by Desojo (2005) the orientation of the acetabulum can be difficult to determine because of crushing of preserved pelves and even more difficult in taxa only known from isolated ilia. Nonetheless various specimens demonstrate that the acetabulum opens ventrally in *Aetosauroides scagliai* (PVL 2073). *Calyptosuchus wellesi* (UMMP 13950), *Scutarx deltatylus* (PEFO 31217), *Typothorax coccinarum* (PEFO 33967), and *Longosuchus meadei* (TMM 31100-236). The acetabulum opens mostly laterally in *Desmatosuchus spurensis* (MNA V9300) and in *Neoaetosauroides engaeus* (PVL 3525). The acetabulae of *Stagonolepis robertsoni* (Walker, 1961) and *Aetosaurus ferratus* (Schoch, 2007) to open ventrally (Desojo, 2005).

50. Pubis, proximal portion, number of 'obturator foramina': one (0); two (1).

Modified from Heckert & Lucas (1999), character 25. Figure 10b.

Walker (1961) described two pubic foramina for *Stagonolepis robertsoni*, which differed from the condition in all other known aetosaurs. However, the pubis of *Scutarx deltatylus* (PEFO 31217) also has two foramina. This character was not considered by Parker (2007) who noted that two foramina were only known for a single taxon (*Stagonolepis robertsoni*) and thus the character was parsimony-uninformative; however, the discovery of that character state in a second taxon (*Scutarx deltatylus*) necessitates reinstatement of the character.

51. Pubis, symphysis length: long, more than one-half of the element length (0); short, less than one-half of the element length (1). New character. Figure 10c.

The pubis symphysis in *Aetosaurus ferratus* (Schoch, 2007) and *Typothorax coccinarum* (Long & Murry, 1995) is short, with the symphysis length less than half the length of the pubis. In contrast, the symphysis in *Desmatosuchus spurensis* (MNA V9300) is long, much more than half the element length (Parker, 2008).

52. Osteoderms, dorsal carapace, transverse smooth strip along anterior edge of osteoderm (= anterior bar): absent (0); present but strongly raised (deliminated from remainder of osteoderm by a distinct trough) (1); present, but weakly raised (2); absent, depressed lamina present instead (3). Modified from Long & Ballew (1985): Table 1. Figure 11a, c-d, f-g, i.

Aetosaurians possess a smooth, narrow, transverse area along the anterior edge of osteoderms that represent the articular surface for slight overlap of the next anteriorly situated osteoderm. When this area is raised it is termed an “anterior bar” (Long & Ballew, 1985). According to those authors, a fully (strongly) raised anterior bar is delimited by a distinct trough along the posterior margin of the smooth area. In *Paratypothorax*, this distinct trough is lacking and the anterior bar is considered to be only weakly raised (Long & Ballew, 1985). In *Desmatosuchus*, the smooth articular surface is depressed below the level of the rest of the ornament surface. This is considered to be an anterior ‘lamina’ rather than an anterior bar (Long & Ballew, 1985).

53. Paramedian osteoderms (any), patterning of dorsal surface: random, no observable pattern (0); radiate (1); reticulate (2); smooth or flat (3). Modified from Long & Ballew (1985): Table 1. Figures 11f-g, j, l.

Long & Ballew (1985) first clearly defined the utility of the surface ornamentation of paramedian osteoderms for aetosaurian taxonomy, and Heckert, Hunt & Lucas (1996) were this first to quantify this as a phylogenetic character. Heckert & Lucas (1999) divided this character into three based on discrete carapace regions (i.e., cervical, dorsal, lateral), but Harris, Gower & Wilkinson (2003) noted that there was no difference in the patterns of these areas and thus reductive coding of this character improperly weighted its’ significance in the analysis. Following this, Parker (2007) devised a composite coding, utilizing only a single character to capture this variation.

In past studies (Heckert, Hunt & Lucas, 1996; Heckert & Lucas, 1999; Parker, 2007) the ornament patterns in *Desmatosuchus* and *Typothorax* have both been considered to be ‘random’ as they lack a clear radial patterning. However, the ornamentation pattern is very different between these taxa with the ornamentation in *Typothorax* consisting mainly of uniformly placed small pits, surrounded by a nearly symmetrical latticework of raised areas, and that of *Desmatosuchus* consisting of pits and grooves of various sizes, offset by raised ridges. The patterning in *Typothorax coccinarum* is described as reticulate or equally spaced, non-radiate pits (Desojo, 2005). Thus a new character state, reticulate, has been added to capture this variation and for scoring the nearly symmetrical pattern found in *Typothorax*.

54. Paramedian osteoderms, dorsal eminence (or center of ossification) position throughout the carapace does not contact the posterior margin of the osteoderm in most rows (0); contacts posterior margin of osteoderm in most osteoderm rows (1). Modified from Long & Ballew (1985), Table 1. Figures 11a-d, f-h, l.

In most aetosaurians the dorsal eminence contacts the posterior plate margin

(e.g., *Calyptosuchus wellesi*); however, in *Desmatosuchus* (e.g., MNA V9300) the boss is just posterior to the center of the osteoderm. In *Paratypothorax*, the boss position varies from close to the center of the osteoderm as in *Desmatosuchus*, to just anterior of the posterior plate margin, to actually contacting the posterior plate margin. This variation is related to anteroposterior position within the carapace, with the bosses being smaller and more anteriorly situated in more anterior osteoderms. The boss migrates posteriorly backwards through the carapace reaching the posterior edge in the anterior caudal/posterior dorsal trunk paramedians. Nonetheless a boss that does not contact the posterior plate margin is more common in dorsal trunk paramedians of *Paratypothorax*, so they are scored as state 1.

The more forward situated eminence in *Paratypothorax* has caused confusion with some workers attempting to assign incomplete osteoderms to specific taxa. For example, all of the paramedian osteoderms from Petrified Forest National Park assigned to *Desmatosuchus* by Long & Ballew (1985) are actually incomplete osteoderms of *Paratypothorax* sp. The key difference is that the dorsal eminence is strongly offset medially in *Paratypothorax*, whereas the boss is centralized in *Desmatosuchus*. In the osteoderms assigned by Long & Ballew (1985) the lateral edges of the osteoderms are missing, making it appear that the bosses are located more centrally than they actually are.

55. Paramedian osteoderms, posterior margin: osteoderm maintains similar thickness throughout posterior to the anterior bar (0); posterior margin bears a transverse, posteroventrally sloping flange (bevel) (1). New character. Figure 11b, i, l.

Dorsal trunk paramedian osteoderms of *Tecovasuchus chatterjeei* (TTU P-00545) and *Paratypothorax andressorum* (SMNS uncatalogued – L10) have a distinct beveling of the posterior plate margin, which is extremely well-developed in *Tecovasuchus chatterjeei* (Martz & Small, 2006).

56. Paramedian osteoderms, transverse anteroposterior thickening (ventral keel or strut) absent, ventral surface is flat (0); weakly developed (1); strongly developed (2). Modified from Heckert, Hunt & Lucas (1996), character 11. Figures 12a-c.

Dorsal trunk paramedian osteoderms with a ventral keel or strut bear a prominent mediolateral thickening of the ventral surface. The keel is strongly developed in *Typothorax coccinarum* (e.g., AMNH FR 2709) where it more than doubles the thickness of the osteoderm. Indeed, the first descriptions of *Typothorax coccinarum* interpreted this strut as a dorsal rib (e.g., Cope 1887; Huene 1915). There is a reduced strut in *Calyptosuchus wellesi* (UCMP 136744; Martz, 2002), and in *Adamanasuchus eisenhardtae* (PEFO 34638). In other taxa, such as *Desmatosuchus spurensis* (MNA V9300), the ventral surface of the osteoderm is completely flat and there is no strut.

57. Paramedian osteoderms, dorsal to the cervical and anterior trunk vertebrae, relative dimensions: wider than long (0); longer than wide (1). Heckert, Hunt & Lucas (1996), character 1. Figures 11a-b.

Aetosaurian cervical dorsal paramedian osteoderms are either rectangular (wider than long) or roughly square (longer than wide, with the anterolateral corner ‘cut off’). Osteoderms that are longer than wide are a synapomorphy of Desmatosuchinae (Parker, Stocker & Irmis, 2008); however, the newly described NCSM 21723, which possesses many desmatosuchine characters, has cervical paramedian osteoderms that are wider than long (Heckert et al., 2015).

58. Paramedian osteoderms, dorsal to the cervical vertebrae (=cervical paramedian osteoderms), ratio of number of osteoderms to number of cervical vertebrae: approximately 1:1 (0); significantly less than 1:1 (1). New character.

Traditionally it was thought that aetosaurians with wider than long cervical paramedian osteoderms (character 56), possessed one osteoderm per cervical vertebra (generally nine), and that aetosaurians with longer than wide osteoderms only had about five or six osteoderms per for the nine vertebrae. However, *Typothorax coccinarum*, which possesses cervical paramedians that are wider than long, only had about five sets of osteoderms covering the entire cervical series (Heckert et al., 2010). This is similar to the counts in *Desmatosuchus* and *Longosuchus*, which have cervical paramedian osteoderms that are longer than wide and only five or six osteoderm sets over the cervical vertebrae.

59. Osteoderms dorsal to the cervical and anterior trunk vertebrae, articular surfaces: adjacent paramedian and lateral osteoderms are separate (0); adjacent paramedian and lateral osteoderms are often fully fused (1). New character. Figures 12c, d, g.

In *Desmatosuchus*, the dorsal and lateral cervical osteoderms are often completed fused (Parker, 2008). Lack of recognition of this fusion caused problems with the identification of some of these osteoderms in past studies (e.g., Brady, 1958:fig. 3; Long & Ballew, 1985: fig 6a). Fusion of the lateral and paramedian cervical osteoderms also occurs in *Longosuchus meadei* (Parker & Martz, 2010).

60. Paramedian osteoderms dorsal to the cervical and anterior trunk vertebrae, lateral edge articulation with lateral osteoderms: vertical 'flat' contact with some interdigitation (0); dorsoventrally thickened, angled contact, with deeply incised interdigitation (='tongue and groove') (1). Modified from Heckert & Lucas (1999), character 46. Figure 11k.

The thickened and complex medial and lateral articular surfaces between osteoderms in *Desmatosuchus* was first described as ‘tongue and groove” by Long & Ballew (1985) and this has been followed by all subsequent workers. Long & Ballew (1985) described this articular surface as greatly thickened (dorsoventrally), strongly rugose and concave. This differs significantly from the thinner, vertical, and generally smoother articular surfaces found in other aetosaurs. This character is presently found only in both species of *Desmatosuchus*, *Longosuchus meadei*, *Lucasuchus hunti*, and *Sierritasuchus macalpini* and was considered a synapomorphy of Desmatosuchinae (Parker, Stocker & Irmis, 2008). The recently described *Gorgetosuchus pekinensis* was described as potentially being closely related to Desmatosuchinae; however, the original describers (Heckert et al., 2015) do not mention if the osteoderm articular surfaces have a ‘tongue and groove’ articular surface, so it is scored here as unknown.

61. Paramedian osteoderms dorsal to the cervical vertebrae, dorsal eminence shape: smooth, not raised above osteoderm surface (0); low, pyramidal or rounded boss or elongate keel (1); tall, cone-shaped in posterior view (2). New character. Figure 11a.

*Longosuchus meadei* (TMM 31185-97), *Desmatosuchus smalli* (TTU P-9024), and *Lucasuchus hunti* (e.g., TMM 31185-60) have cervical paramedian osteoderms which bear prominent raised dorsal eminences. In contrast, in *Aetosauroides scagliai* (PVL 2073) and *Stagonolepis robertsoni* (Walker, 1961) have dorsal eminences that are barely visible and in *Typothorax coccinarum* (e.g., NMMNH P-56299), dorsal eminences are not visible until well into the dorsal trunk paramedian series (Heckert et al., 2010).

62. Osteoderms dorsal to the trunk vertebrae, articulation of lateral and paramedian osteoderms: the anterior edge of paramedian osteoderm overlaps the anterior edge of lateral osteoderm (0); the anterior edge of lateral osteoderm overlaps the anterior edge of paramedian osteoderm (1). Parker & Martz (2010). Figures 11g-h.

Aetosaurians have two distinct articulation patterns between the anterior edges of the paramedian osteoderm and the corresponding lateral osteoderm. In *Longosuchus meadei* (TMM 81185-84B) the anterolateral corner of the paramedian osteoderm has a dorsally facing articular facet that in articulation is overlapped by the anteromedial corner of the lateral osteoderm. In *Scutarx deltatylus* (e.g., PEFO 34616) the anteromedial corner of the lateral osteoderm is rounded and has a dorsally facing facet that receives the anteromedial projection of the paramedian osteoderm.

63. Paramedian osteoderms dorsal to the trunk vertebrae, “shape of the lateral edge in dorsal view: roughly straight or sigmoidal (0); sigmoidal with strongly posteromedially oriented posterolateral ("cut-off") corner (1). New character. Figures 11d, f-g, i.

*Stagonolepis robertsoni* (Walker, 1961), *Desmatosuchus spurensis* (MNA V9300), and *Aetosaurus ferratus* (Schoch, 2007) possess dorsal trunk paramedian osteoderms with roughly straight to sigmoidal lateral edges in dorsal view. *Paratypothorax* sp. (PEFO 3004) and *Adamanasuchus eisenhardtae* (PEFO 34638) possess strongly sigmoidal lateral edges in dorsal view, with a posterolateral corner that appears to have been sheared off. The strongly posteromedially oriented edge corresponds with a prominent triangular posterolateral projection of the lateral plate that fills the space voided by the ‘cut-off corner’ of the paramedian in the dorsal carapace.

64. Paramedian osteoderms dorsal to the trunk vertebrae, width/length ratio of widest osteoderms (rows 9-11) in series: less than 3.0 (0); 3.01-3.5 (1); 3.5 or more (2).

Modified from Parrish (1994), character 15. Figures 11d, f-g.

As originally constructed by Parrish (1994) this character had two states, width/length ratio greater than or equal to 4.0 and width/length ratio less than 4.0 (Heckert, Hunt & Lucas, 1996; Heckert & Lucas, 1999). Parker (2007) argued that only *Paratypothorax* possessed a width/length ratio greater than 4.0 so he reduced the division point to 3.5 to make the character more applicable. Heckert & Lucas (1999) coded *Typothorax coccinarum* as possessing a maximum width/length ratio of greater than 4.0; however, in the course of this study a paramedian osteoderm of *Typothorax* with that great of a ratio was not observed. The widest paramedian osteoderm from the UCMP Canjilion Quarry material has a ratio of about 3.88 and the Revuelto Creek *Typothorax* has a maximum of 3.5 (Heckert et al., 2010). Even the extremely large paramedian plate (PEFO 23388) discussed by Parker and Irmis (2005) falls short of having a 4.0 ratio. However, referred material of *Redondasuchus rineharti* at the MCCDM (field number 2011RRBWKB#9) has a ratio of around 4.5 (J. Martz, pers. comm., 2013).

Most aetosaurs fall within the 3.01-3.5 ratio range; however a few have maximum ratios of less than 3.0. These include *Longosuchus meadei*, *Desmatosuchus spurensis*, *Stagonolepis robertsoni*, and *Aetobarbakinoides brasiliensis*.

65. Paramedian osteoderms dorsal to the trunk vertebrae; posteromedial surface of osteoderm ornamentation: lacking distinct transverse ridge between dorsal eminence and medial margin of the osteoderm. (0); distinct sharp raised mediolateral ridge extends medially from dorsal eminence to medial osteoderm margin (1). New character. Figures 11b, i.

The dorsal trunk paramedian osteoderms of *Tecovasuchus chatterjeei* (TTU P-545) bear a distinct mediolateral ridge that originates at the dorsal eminence and extends medially to contact the posteromedial plate margin. This ridge delineates the posterior beveled area in *Tecovasuchus chatterjeei*. This ridge is also present in *Paratypothorax* sp. (TTU P-9169), and although faint is also present in *Paratypothorax andressorum* (SMNS uncatalogued, R12).

66. Paramedian osteoderms dorsal to the trunk vertebrae, position of dorsal eminence relative to the center of the osteoderm centralized (0); moderately offset medially (1); strongly offset medially (2). Parker (2007), character 29. Figures 11f-g, j.

In *Desmatosuchus spurensis* (UMMP 7476) the dorsal eminence is situated centrally on the mediolateral axis of the paramedian osteoderm. It is shifted medially in *Stagonolepis robertsoni* (Walker, 1961), and it is shifted even further medially, almost to the medial edge of the osteoderm, in *Paratypothorax andressorum* (Long & Ballew, 1985)

67. Paramedian osteoderms dorsal to the trunk vertebrae, anterior margin of the anteromedial corner of the anterior bar in dorsal view: anteriorly directed triangular projection (0); straight (1). New Character. Figures 11d, g.

In aetosaurians such as *Stenomyti huangae* (Small & Martz, 2013), *Stagonolepis robertsoni* (Walker, 1961), and *Paratypothorax andressorum* (SMNS uncatalogued, L16) the anteromedial corner of the anterior bar bears a sharp triangular anterior projection. This projection is lacking in other aetosaurs such as *Desmatosuchus spurensis* (MNA V9300) where the anteromedial corner of the anterior bar is mediolaterally straight.

68. Paramedian osteoderms dorsal to the trunk vertebrae, lateral margin of the anterolateral corner of the anterior bar: distinct triangular lateral projection that barely extends beyond the lateral osteoderm margin (projection may be rounded distally (0); distinct triangular lateral projection that extends well beyond the lateral osteoderm margin (1); corner embayed for reception of the anteromedial projection of the lateral osteoderm (2). New character. Figures 11d, i, l.

Many non-desmatosuchine aetosaurs possess dorsal trunk paramedian osteoderms with anterior bars that bear an anterolateral projection that extends beyond that lateral margin of the main plate body. The length and distal end shape of the process are variable. *Aetosauroides scagliai* (PVL 2073) possesses a projection that is mediolaterally short with a pointed distal end. *Scutarx deltatylus* (PEFO 34616) also possesses a pointed process that is significantly more elongate. *Paratypothorax* sp. (PEFO 3004) has the shorter process, which instead of being pointed, it is gently rounded in dorsal view. This condition is coded the same as the short, pointed process as this character is based on process length and not process end shape. These anterolateral projections fill in a void in the anteromedial corner of the adjacent lateral osteoderm, slightly overlapping that portion of the lateral osteoderm.

69. Paramedian osteoderms dorsal to the trunk vertebrae, anterior margin of anterior bar/lamina on the medial side of the osteoderm: anteriorly concave ('scalloped") (0); straight (1). New character. Figures 11d, g, j.

The anterior bar in many aetosaurians bear a ‘scalloped-out’ anterior margin on the medial side of the dorsal trunk paramedian osteoderm. In plates with this character the anterior edge of the anterior bar is concave in dorsal view with the bar thinning anteroposteriorly in the center of the medial portion of the bar. Often this scalloping is associated with a triangular anterior process of the anterior bar that is directly anterior to the dorsal eminence. This character state is very prominent in the dorsal trunk paramedians of *Stagonolepis robertsoni* (Walker, 1961) and *Scutarx deltatylus* (PEFO 34045). It is absent in *Desmatosuchus spurensis* (MNA V9300) and *Longosuchus meadei* (TMM 31185-97). In *Typothorax coccinarum* (e.g., UCMP 34227), the medial portion of the anterior bar thins drastically, but the anterior projection anterior to the dorsal eminence does not appear to be present.

70. Paramedian osteoderms dorsal to the trunk vertebrae, posteromedial corner: flat with ornamentation (0); flat triangular area devoid of ornamentation (1); prominent raised triangular tuberosity devoid of ornamentation (2). [Ordered]. New character. Figures 11l, 12e-f.

An apparent autapomorphy of *Scutarx deltatylus* (e.g., PEFO 34616) is the presence of a large triangular protuberance in the posteromedial corner of the dorsal trunk paramedian osteoderms. However, although *Adamanasuchus eisenhardtae* (PEFO 34638) lacks the tuberosity, a triangular area in the same position is devoid of ornamentation, and this is considered a variation of this character.

71. Paramedian osteoderms dorsal to the anterior caudal vertebrae, dorsal eminence shape: absent, no dorsal eminence (0); low and pyramidal or rounded and knob-like (1); moderate, bulbous spike (2); tall, cone-shaped (3); tall anteriorly curved elongate spine (4). Modified from Long & Ballew (1985): Table 1. Figures 11c-e, g, j.

All aetosaurians, with the possible exception of *Redondasuchus reseri*, possess a raised dorsal eminence (boss) on the dorsal surface of the paramedian plates. In most taxa this boss takes the form of a low pyramidal or rounded knob, but in others the eminence is dorsoventrally taller. Taller eminences take three distinct forms. In *Paratypothorax* they have the shape of a bulbous spike (although they tend to be larger in the German specimens). In *Lucasuchus hunti* (TMM 31100-361) , they are in the form of what Long & Murry (1995) described as an ‘inverted ice-cream cone’ in that they are extremely tall and conical with a broad rounded base. The third form is found in *Rioarribasuchus chamaensis* (e.g., NMMNH P-32793), where the dorsal eminence in the posterior trunk and anterior pelvic areas is an elongate, gracile, anteromedially recurved spine. Presently each of these tall forms is found in separate taxa. I do not combine them into a single ‘tall’ character because although the presence of the boss itself is homologous, it is not clear that a tall boss is homologous because it has taken so many unique forms.

72. Lateral osteoderms: absent (0); present (1). New character.

All known aetosaurs preserve lateral osteoderms with the exception of *Redondasuchus reseri* (Hunt & Lucas, 1991) and *Aetobarbakinoides brasiliensis* (Desojo, Ezcurra & Kischlat, 2012); however, despite this absence, the evidence that they completely lacked lateral osteoderms is unequivocal. Lateral osteoderms are not present in *Postosuchus kirkpatricki* (Nesbitt, 2011; Weinbaum, 2013). Presently this character offers no ingroup resolution, but is included for future studies of aetosaurians and their closest relatives.

73. Lateral osteoderms, distribution within carapace; absent (0); only present in the sacral and anterior caudal region (1); present through the entire carapace (2). New character.

In all aetosaurians that preserve lateral armor this osteoderms extend through the entire carapace and even in taxa with incomplete carapaces there is no evidence to the contrary. However, *Revueltosaurus callenderi* almost certainly had lateral osteoderms in the pelvic region (W. Parker, unpublished data). Presently this character offers no ingroup resolution, but is included for future studies of aetosaurians and their closest relatives.

74. Lateral osteoderms dorsolateral to the cervical vertebrae, form of the dorsal eminence: low keel or knob (0); moderate length dorsoventrally flattened slightly recurved spine (1); moderate length faceted, slightly recurved spine (2); greatly elongated horn (3). Figures 12g; 13h.

Taxa such as *Aetosaurus ferratus* (Schoch, 2007) and *Coahomasuchus kahleorum* (Heckert & Lucas, 1999) possess cervical lateral osteoderms bearing dorsal eminences in the form of a low keel or knob. In other taxa such as *Longosuchus meadei* (TMM 31185-84B) and *Paratypothorax* sp. (VRPH 2) the eminence takes the form of a moderately elongate spine; however, in the former the spine is distinctly faceted. The facets, *sensu* Hunt & Lucas (1990), provide the spine with a trihedral cross-section (Lucas, 1998). The extreme is seen in *Desmatosuchus* (e.g., UMMP 7476) where the eminence is an enormous posteriorly recurved horn.

75. Lateral osteoderms dorsolateral to the anterior trunk vertebrae, dorsal eminence form knob or spine (0); large and hemispherical (1). Modified from Parker (2007), character 37. Figure 13e.

In *Desmatosuchus* the dorsal eminences on the first three dorsal trunk lateral osteoderms, situated just posterior to the large hornlike dorsal eminence of the posterior cervical laterals, are in the forms of a large, but low, ovate mounds (Brady, 1958:fig. 3; Long & Ballew, 1985:fig. 6a; Parker, 2008:figs. 24a-c). Long & Ballew (1985) considered this shape for osteoderms of the pelvic region; however, the articulated specimen MNA V9300 demonstrated that they are instead from the anterior dorsal region (Parker, 2008).

76. Lateral osteoderms dorsolateral to the trunk vertebrae, shape of dorsal flange: broad rectangle (0); triangular (1); highly reduced in size and a narrow triangle ("tongue-shaped") (2). [Ordered]. Parker (2007), character 36. Figures 13b-c, g, i-k.

The dorsal flange of the lateral osteoderms is the portion of the osteoderm that is medial to the dorsal eminence. The lateral flanges of many of the dorsal lateral osteoderms in *Calyptosuchus wellesi* (e.g., UCMP 27225) are distinctly triangular in dorsal view. This differs significantly from the dorsal flanges in *Tecovasuchus chatterjeei* (TTU P-545) and *Paratypothorax* sp. (PEFO 3004), which are much reduced and ‘tongue-like’ (Martz & Small, 2006), as well as the rectangular dorsal flanges of *Desmatosuchus spurensis* (MNA V9300) and *Longosuchus meadei* (TMM 31185-97). The triangular dorsal flange in *Calyptosuchus wellesi*, results from the elongate anterolateral process of the adjacent paramedian osteoderm projecting into the ‘space’ of the lateral osteoderm. Some of the lateral osteoderms of *Calyptosuchus wellesi* are sub-rectangular in dorsal view (e.g., UCMP 27225), however, these co-occur in the same carapace with the triangular osteoderms and differ from taxa such as *Desmatosuchus spurensis* where all of the lateral osteoderms in the carapace bear rectangular flanges. Thus *Calyptosuchus wellesi* is scored for state 1, as at least some of the osteoderm lateral flanges are distinctly triangular.

Although the dorsal trunk lateral osteoderms of *Typothorax coccinarum* (e.g., NMMNH P-56299) share many characters with *Paratypothorax* sp., rather than *Calyptosuchus wellesi* (e.g., strongly acute angle of flexion, ‘blade-like’ flanges forming a curved spine in ventral view), the dorsal flange is clearly triangular in dorsal view and not ‘tongue-like’ as in *Paratypothorax*, thus *Typothorax coccinarum* is scored here as possessing state 1.

77. Lateral osteoderms dorsolateral to the trunk vertebrae, ventralmost 1/3 of the posterior face of the dorsal eminence (spine) ventral margin straight (0); distinct ventrally concave embayment ('emarginated') (1). Heckert & Lucas (1999a), character 48. Figures 12g; 13b, j.

In taxa where the dorsal eminence of the dorsal trunk lateral osteoderms forms a spine, there is a deep ventral, triangular emargination of the posterior face of the spine in some (e.g., *Longosuchus meadei*). This emargination is lacking in other taxa such as *Lucasuchus hunti* (Parker & Martz, 2010). This character is scored as inapplicable for taxa lacking a spine-like dorsal eminence, because absence of the spine is not the same as absence of the ventral emargination.

78. Lateral osteoderms dorsolateral to the mid-trunk region, form of the dorsal eminence: triangular boss or keel, not elongated (0); elongated flattened horn (1); conical spike (2).

Parker (2007), character 30. Figures 13a-d, f-g, i-k.

A number of aetosaurians (e.g., *Calyptosuchus wellesi*, *Aetosaurus ferratus*) lack the extension of the dorsal eminence of the dorsal trunk lateral osteoderms into an elongate conical spike as in *Desmatosuchus spurensis* (UMMP 7476) and *Lucasuchus hunti* (TMM 31185-66). In *Typothorax coccinarum* (e.g., AMNH 2713) and *Paratypothorax andressorum* (SMNS uncatalogued, L18) the dorsal and lateral (ventral) flanges are mediolaterally elongate and meet along an extended transverse edge. This edge continues laterally and terminates in a slightly recurved point. Thus it is more like an elongate flattened horn than a conical spine.

79. Lateral osteoderms dorsolateral to the mid-trunk region, angle of flexion between dorsal and lateral flanges of the osteoderms obtuse (0); approximately 90 degrees (1); strongly acute (2). [Ordered] Modified from Heckert, Hunt & Lucas. (1996), character 14. Figures 14a, d, j.

This character is variable through the anteroposterior extent of the carapace and thus is restricted to the mid-lateral to posterior dorsal trunk lateral osteoderms. Aetosaurians with obtuse angles of flexion between the dorsal and lateral flanges include *Aetosaurus ferratus* (Schoch, 2007) and *Coahomasuchus kahleorum* (Heckert & Lucas, 1999). Heckert & Lucas (1999) considered the dorsal trunk lateral osteoderms of the holotype of *Coahomasuchus kahleorum* (NMMNH P-18496) to be completely flat with not sign of a division into dorsal and lateral flanges. This would be autapomorphic for Aetosauria; however, in NMMNH P-18496, the lateral osteoderms have small, triangular dorsal flanges as in *Aetosaurus ferratus* and *Calyptosuchus wellesi* (UCMP 27225) and flattening of the carapace because of crushing has pushed the dorsal flanges downward and partially obscuring them. However, they can be seen in the holotype (Heckert & Lucas, 1999: fig. 3) on the right side in the waist narrowing and anterior to it. Note also that the width of the lateral flange increases significantly anterior to the waist compared to the very reduced dorsal flange and this helps to create the illusion that these is no discrete dorsal flange. The presence of a discrete triangular flange and obtuse flexion of the lateral osteoderms is also confirmed by isolated osteoderms in TMM 31100-437. Specimens that possess an obtuse angle of flexion retain this through the entire carapace. *Calyptosuchus wellesi* has been described as possessing cervical laterals with an acute angle of flexion (Long & Ballew, 1985; Long & Murry, 1995); however, these are dorsal lateral osteoderms of a ‘*Tecovasuchus*-like’ taxon (Parker, 2005).

*Desmatosuchus spurensis* (e.g., MNA V9300) and *Longosuchus meadei* (TMM 31185-97) possess distinct dorsal and lateral flanges that meet at an angle of around 90 degrees. This varies little throughout the carapace. *Typothorax coccinarum* (e.g., NMMNH P-56299) and *Paratypothorax andressorum* (SMNS unnumbered, L18) have dorsal and lateral flanges that meet at an acute angle and are scored as such; however, other lateral osteoderms in these taxa are anteroposteriorly shorter and have flanges that meet at right angles (e.g., *Paratypothorax andressorum* (SMNS unnumbered, L9, R10). It is not entirely certain where in the carapace this character state occurs but scoring of disarticulated, incomplete material needs to be done with caution. If a specimen possesses the acute angle in at least one osteoderm, I code the taxon as possessing that state even if the angle of flexion in other osteoderms in the same carapace may trend closer to 90 degrees. Completion of preparation and description of SMNS 19003 should reveal these changes in angle of flexion in an aetosaur with the acute osteoderm type.

80. Lateral osteoderms dorsolateral to the mid-trunk region, symmetry of dorsal and lateral flanges: weakly or strongly asymmetrical, with lateral flange the longest (0); strongly asymmetrical with dorsal flange longest (1). Modified from Heckert, Hunt & Lucas (1996), character 15. Figures 14a,c, i.

This character considers size differences (symmetry) of the lateral and dorsal flanges of the dorsal trunk lateral osteoderms as divided by the axis of the dorsal eminence. No aetosaurian lateral osteoderm is perfectly symmetrical; however, in taxa where the symmetry is close the lateral flange is still larger than the dorsal flange (e.g., *Aetosauroides scagliai*). Where the flanges are strongly asymmetrical, some taxa such as *Coahomasuchus kahleorum* (NMMNH P-18496), have greatly reduced dorsal flanges where the dorsal flange is a small triangle and the lateral flange is a wide rectangle (see Heckert & Lucas, 1999: fig. 3). In *Desmatosuchus spurensis* (MNV V9300), the mid-dorsal trunk lateral osteoderms possess elongate dorsal flanges, and reduced lateral flanges (Parker, 2008).

81. Lateral osteoderms dorsolateral to the sacral and anterior caudal vertebrae, lateral flange shape: roughly rectangular and lateral to a sharp medially situated keel (0); roughly triangular in lateral view with a semicircular ventrolateral border and a hook-like eminence (1); rectangular and ventral to a well-developed spine (2). Parker (2007), character 28. Figures 13c, f, j.

This character attempts to capture the anatomical variation in the lateral flange of the pelvic and anterior caudal lateral osteoderms. A roughly triangular osteoderm with a semicircular ventrolateral border and a hook-like eminence is shared by several taxa including *Typothorax coccinarum* (NMMNH P-56299) and *Paratypothorax sp.*(PEFO 3004)*,* and differs strikingly from the spiked plate found in *Desmatosuchus spurensis* (MNA V9300) and *Longosuchus meadei* (TMM 31185-84b)*.* A somewhat similar form occurs in *Aetosaurus ferratus* (Schoch, 2007)*, Stagonolepis robertsoni* (Walker, 1961)*,* *Aetosauroides scagliai* (PVL 2073)*,* and *Neoaetosauroides engaeus* (PVL 3525)*,* but these plates are more rectangular rather than triangular (Parker, 2007).

82. Carapace, overall shape in dorsal view: presence of narrow waist anterior to sacrum (0); moderate spinose carapace (1); broad discoidal carapace (2). Modified from Heckert, Hunt & Lucas (1996), character 16.

The aetosaur carapace comes in three general forms, a narrow carapace with a distinct narrowing (waist) just anterior to the pelvis (e.g., *Aetosaurus ferratus*), a broader carapace that lacks the narrow waist and is generally spinose (e.g., *Desmatosuchus spurensis*), and a very broad discoid form (e.g., *Typothorax coccinarum*) (Desojo et al., 2013:fig. 1).

83. Osteoderms ventral to the trunk vertebrae, shape and arrangement: absent (0); irregular, non-touching (1); square, overlapping (2). New character.

Previous characters regarding the ventral osteoderms focused on number of rows or the type of ornament; however, these were difficult to score given the incomplete preservation of the ventral osteoderms in many taxa. Instead, this new character focuses on the shape and arrangement of the ventral osteoderms in light of the recent discovery of *Stenomyti huangae*, which has a unique arrangement of the ventral osteoderms (Small & Martz, 2013). In aetosaurians such as *Stagonolepis robertsoni* (Walker, 1961) and *Coahomasuchus kahleorum* (Heckert & Lucas, 1999) the associated ventral osteoderms consists of rows and columns of overlapping, equant osteoderms. However, in *Stenomyti huangae* the ventral osteoderms are round to oval and non-overlapping (Small & Martz, 2013). Despite the recovery of several nearly complete skeletons of *Desmatosuchus* and *Longosuchus meadei*, no ventral osteoderms are known for the taxa and it is hypothesized here that these forms lacked ventral armor (Parker, 2008).

##

## REFERENCES

Brady LF. 1958. New occurrence of *Desmatosuchus* in Northern Arizona. *Plateau* 30:61-63.

Casamiquela RM. 1961. Dos nuevos estagonolepoideos Argentinos (de Ischigualasto, San Juan). *Revista de la Asociación Geológica de Argentina* 16:143-203.

Casamiquela RM. 1967. Materiales adicionales y reinterpretación de *Aetosauroides scagliai* (de Ischigualasto, San Juan). *Revista del Museo de La Plata (nueva serie), Tomo 5, Sección Paleontología* 33:173-196.Desojo JB. 2005. Los aetosaurios (Amniota, Diapsida) de América del Sur: sus relaciones y aportes a la biogeografía y bioestratigrafía del Triásico continental. *PhD thesis*, Universidad de Buenos Aires.

Case EC. 1922. New reptiles and stegocephalians from the Upper Triassic of western Texas. *Carnegie Institution of Washington Publication* 321:1-84.

Desojo JB, Báez AM. 2005. El esqueleto postcraneano de *Neoaetosauroides* (Archosauria: Aetosauria) del Triásico Superior del centro-oeste de Argentina. *Ameghiniana* 42:115-126.

Desojo JB, Báez AM. 2007. Cranial morphology of the Late Triassic South American archosaur *Neoaetosauroides engaeus*: evidence for aetosaurian diversity. *Palaeontology* 50:267-276.

Desojo JB, Vizcaíno SF. 2009. Jaw biomechanics in the South American aetosaur *Neoaetosauroides engaeus*. *Paläontologische Zeitschrift* 83:499–510.

Desojo JB, Ezcurra MD. 2011. A reappraisal of the taxonomic status of *Aetosauroides* (Archosauria, Aetosauria) specimens from the Late Triassic of South America and their proposed synonymy with *Stagonolepis*. *Journal of Vertebrate Paleontology* 31:596-609.

Desojo JB, Schoch RR. 2014. Cranial anatomy of the large aetosaur *Paratypothorax andressorum* from the Upper Triassic of Germany [Abstract]. *Journal of Vertebrate Paleontology, Program and Abstracts 2014*:120.

Desojo JB, Ezcurra MD, Kischlat EE. 2012. A new aetosaur genus (Archosauria: Pseudosuchia) from the early Late Triassic of southern Brazil. *Zootaxa* 3166:1-33.

Desojo JB, Heckert AB, Martz JW, Parker WG, Schoch RR, Small BJ, Sulej T. 2013. Aetosauria: a clade of armoured pseudosuchians from the Upper Triassic continental beds. In: Nesbitt SJ, Desojo JB, Irmis RB, eds. *Anatomy, Phylogeny, and Palaeobiology of Early Archosaurs and their Kin*. Geological Society, London, Special Publications 379. Bath: The Geological Society Publishing House. 203-239.

Harris SR, Gower DJ, Wilkinson M. 2003. Intraorganismal homology, character construction, and the phylogeny of aetosaurian archosaurs (Reptilia, Diapsida). *Systematic Biology* 52:239-252.

Heckert AB, Lucas SG. 1999. A new aetosaur (Reptilia: Archosauria) from the Upper Triassic of Texas and the phylogeny of aetosaurs. *Journal of Vertebrate Paleontology* 19:50-68.

Heckert AB, Lucas SG. 2002. South American occurrences of the Adamanian (Late Triassic: latest Carnian) index taxon *Stagonolepis* (Archosauria: Aetosauria) and their biochronological significance. *Journal of Paleontology* 76:852-863.

Heckert AB, Hunt AP, Lucas SG. 1996. Redescription of *Redondasuchus reseri*, a Late Triassic aetosaur (Reptilia: Archosauria) from New Mexico (U.S.A.), and the biochronology and phylogeny of aetosaurs. *Geobios* 29:619-632.

Heckert AB, Lucas SG, Rinehart LF, Celeskey MD, Spielmann JA, Hunt AP. 2010. Articulated skeletons of the aetosaur *Typothorax coccinarum* Cope (Archosauria: Stagonolepididae) from the Upper Triassic Bull Canyon Formation (Revueltian: early-mid Norian), eastern New Mexico, USA. *Journal of Vertebrate Paleontology* 30:619-642.

Heckert AB, Schneider VP, Fraser NC, Webb RA. 2015. A new aetosaur (Archosauria: Suchia) from the Upper Triassic Pekin Formation, Deep River Basin, North Carolina, U. S. A., and its implications for early aetosaur evolution. *Journal of Vertebrate Paleontology* 35:e881831.

Huene Fv. 1915. On reptiles of the New Mexican Trias in the Cope collection. *American Museum of Natural History Bulletin* 34:485-507.

Hunt AP, Lucas SG. 1990. Re-evaluation of ‘*Typothorax*’ *meadei*, a Late Triassic aetosaur from the United States. *Paläontologische Zeitschrift* 64:317-328.

Landry, SO. 1958. The function of the entepicondylar foramen in mammals. *American Midland Naturalist* 60:100-112.

Long RA, Ballew KL. 1985. Aetosaur dermal armor from the late Triassic of southwestern North America, with special reference to material from the Chinle Formation of Petrified Forest National Park. In: Colbert EH, Johnson RR, eds. *The Petrified Forest Through the Ages, 75^th^ Anniversary Symposium November 7, 1981*. Museum of Northern Arizona Bulletin 54. Flagstaff: Museum of Northern Arizona Press. 45-68.

Long RA, Murry PA. 1995. Late Triassic (Carnian and Norian) tetrapods from the southwestern United States. *New Mexico Museum of Natural History and Science Bulletin* 4:1–254.

Lucas SG. 1998. The aetosaur *Longosuchus* from the Triassic of Morocco and its biochronological significance. *Comptes rendus de l’Académie des science Série II, sciences de la terre et des planètes* 326:589–594.

Lucas SG, Heckert AB. 2001. The aetosaur *Stagonolepis* from the Upper Triassic of Brazil and its biochronological *significance. Neues Jahrbuch für Geologie und Paläontologie, Monatshefte* 2001:719–732.

Martz JW. 2002. The morphology and ontogeny of *Typothorax coccinarum* (Archosauria, Stagonolepididae) from the Upper Triassic of the American Southwest. *MS thesis*, Texas: Texas Tech University, Lubbock. 279.

Martz JW, Small BJ. 2006. *Tecovasuchus chatterjeei*, a new aetosaur (Archosauria: Aetosauria) from the Tecovas Formation (Upper Triassic, Carnian) of Texas. *Journal of Vertebrate Paleontology* 26:308–320.

Murry PA, Long RA. 1996. A diminutive carnivorous aetosaur from the Upper Triassic of Howard County, Texas [Abstract]. *Journal of Vertebrate Paleontology* 16 (supplement to number 3):55A.

Nesbitt SJ. 2011. The early evolution of archosaurs: relationships and the origin of major clades. *Bulletin of the American Museum of Natural History* 352:1–292.

Parker WG. 2005a. Faunal review of the Upper Triassic Chinle Formation of Arizona. In: McCord RD, ed. *Vertebrate Paleontology of Arizona*. Mesa Southwest Museum Bulletin 11. Mesa: Southwest Paleontological Society, Mesa Southwest Museum and the City of Mesa. 34-54.

Parker WG. 2007. Reassessment of the aetosaur “*Desmatosuchus*” *chamaensis* with a reanalysis of the phylogeny of the Aetosauria (Archosauria: Pseudosuchia). *Journal of Systematic Palaeontology* 5:1–28.

Parker WG. 2008b. Description of new material of the aetosaur *Desmatosuchus spurensis* (Archosauria: Suchia) from the Chinle Formation of Arizona and a revision of the genus *Desmatosuchus*. *PaleoBios new series* 28:1–40.

Parker WG, Irmis RB. 2005. Advances in Late Triassic vertebrate paleontology based on material from Petrified Forest National Park, Arizona. In: Heckert AB, Lucas SG, eds. *Vertebrate Paleontology in Arizona*. New Mexico Museum of Natural History and Science Bulletin 29. Albuquerque: New Mexico Museum of Natural History and Science. 45-58.

Parker WG, Martz JW. 2010. Using positional homology in aetosaur (Archosauria: Pseudosuchia) osteoderms to evaluate the taxonomic status of *Lucasuchus hunti*. *Journal of Vertebrate Paleontology* 30:1100–1104.

Parker WG, Martz JW. 2011. The Late Triassic (Norian) Adamanian–Revueltian tetrapod faunal transition in the Chinle Formation of Petrified Forest National Park, Arizona. In Butler RJ, Irmis RB, Langer MC, Smith AB, eds. *Late Triassic Terrestrial Biotas and the Rise of Dinosaurs*. Earth and Environmental Science Transactions of the Royal Society of Edinburgh 101 (for 2010). 231-260.

Parker WG, Stocker MR, Irmis RB. 2008. A new desmatosuchine aetosaur (Archosauria: Suchia) from the Upper Triassic Tecovas Formation (Dockum Group) of Texas. *Journal of Vertebrate Paleontology* 28:692–701.

Parrish JM. 1994. Cranial osteology of *Longosuchus meadei* and the phylogeny and distribution of the Aetosauria. *Journal of Vertebrate Paleontology* 14:196-209.

Roberto-Da-Silva L, Desojo JB, Cabriera SF, Aires ASS, Müller ST, Pacheco CP, Dias-Da-Silva S. 2014. A new aetosaur from the Upper Triassic of the Santa Maria Formation, southern Brazil. *Zootaxa* 3764:240-278.

Romer AS. 1956. *Osteology of the Reptiles*. Chicago: University of Chicago Press. 772.

Schoch RR. 2007. Osteology of the small archosaur *Aetosaurus* from the Upper Triassic of Germany. *Neues Jahrbuch für Geologie und Paläontologie, Abhandlungen* 246:1–35.

Small BJ. 2002. Cranial anatomy of *Desmatosuchus haplocerus* (Reptilia: Archosauria: Stagonolepididae). *Zoological Journal of the Linnean Society* 136:97-111.

Small BJ, Martz JW. 2013. A new basal aetosaur from the Upper Triassic Chinle Formation of the Eagle Basin, Colorado, USA. In Nesbitt SJ, Desojo JB, Irmis RB, eds. *Anatomy, Phylogeny and Palaeobiology of Early Archosaurs and their Kin*. Geological Society, London, Special Publications 379. Bath: The Geological Society Publishing House. 393-412.

Sulej T. 2010. The skull of an early Late Triassic aetosaur and the evolution of the stagonolepidid archosaurian reptiles. *Zoological Journal of the Linnean Society* 158:860-881.

Walker AD. 1961. Triassic Reptiles from the Elgin Area: *Stagonolepis*, *Dasygnathus*, and their allies. *Philosophical Transactions of the Royal Society of London* 244:103–204.

Weinbaum JC. 2011. The skull of *Postosuchus kirkpatricki* (Archosauria: Paracrocodyliformes) from the Upper Triassic of the United States. *PaleoBios new series* 30:18-44.

Weinbaum JC. 2011. The skull of *Postosuchus kirkpatricki* (Archosauria: Paracrocodyliformes) from the Upper Triassic of the United States. *PaleoBios new series* 30:18-44.

Weinbaum JC. 2013. Postcranial skeleton of *Postosuchus kirkpatricki* (Archosauria: Paracrocodylomorpha) from the Upper Triassic of the United States. In: Nesbitt SJ, Desojo JB, Irmis RB, eds. *Anatomy, Phylogeny and Palaeobiology of Early Archosaurs and their Kin*. Geological Society, London, Special Publications 379. Bath: The Geological Society Publishing House. 525-553.

Wilson JA. 1999. Vertebral laminae in sauropods and other saurischian dinosaurs. *Journal of Vertebrate Paleontology* 19:639-653.

Figure Captions

Figure 1: Skull reconstructions of suchian archosaurs showing defined character states. A, B, C, *Desmatosuchus smalli* in lateral, dorsal and ventral views (redrawn from Small, 2002); D, E, F, *Stenomyti huangae* in lateral, dorsal, and ventral views (redrawn from Small and Martz, 2013); G, *Aetosauroides scagliai* in lateral view (redrawn from Desojo and Ezcurra, 2011); H, *Revueltosaurus callenderi* (based on PEFO 34561) in lateral view. Scale bars equal 5 cm.

Figure 2: Photos of aetosaurian crania showing defined character states. A, close-up view of the antorbital fenestra in SMNS 19003, showing the extent of the antorbital fossa and the upper contact with the frontal; B, skull of *Aetosauroides scagliai* (PVL 2073) in dorsal view. Scale bars equal 1 cm.

Figure 3: Photos of aetosaurian basicrania showing defined character states. A, PVSJ 326, parabasisphenoid of *Aetosauroides scagliai* in ventral view; B, TTU P-9024, parabasisphenoid of *Desmatosuchus smalli* in ventral view; C, UCMP 27409, parabasisphenoid of an aetosaurian, possibly *Calyptosuchus wellesi,* in ventral view; Scale bars equal 1 cm.

Figure 4: Posterior portion of the left mandible of *Stagonolepis olenkae* (ABIII 578/34) in lateral view showing defined character states. Scale bar equal 1 cm.

Figure 5: Maxillary teeth of various aetosaurians and *Revueltosaurus callenderi* showing defined character states. A, SMNS 19003; B, *Revueltosaurus callenderi* (PEFO 34561); C, *Desmatosuchus smalli* (TTU P-9024); D, *Coahomasuchus kahleorum* (TMM 31100-437). Scale bars equal 1 cm.

Figure 6: Cervical series centra of aetosaurians showing defined character states. A, *Desmatosuchus spurensis* (UMMP 7504) in anterior view; B, *Redondasuchus rineharti* (MDM 20080809BDM006RRF 34561) in lateral view; C, *Sierritasuchus macalpini* (UMMP V60817) in ventral view; D, *Calyptosuchus wellesi* (UMCP 139837) in posterior view; E, *Calyptosuchus wellesi* (UCMP 139794) in lateral view; F, *Calyptosuchus wellesi* (UCMP 78714) in ventral view. Scale bars equal 1 cm.

Figure 7: Dorsal and caudal series vertebrae of aetosaurians showing defined character states. A, *Desmatosuchus spurensis* (MNA V9300) anterior dorsal vertebra in lateral view; B, *Typothorax coccinarum* (TTU P-09214) posterior dorsal vertebra in anterior view; C, *Calyptosuchus wellesi* (UMCP 139702) mid-dorsal vertebra in anterior view; D, *Aetosauroides scagliai* (PVL 2073) dorsal vertebrae in lateral view; E, *Paratypothorax* sp. (PEFO 3004) anterior caudal vertebra in anterior view; F, *Desmatosuchus spurensis* (MNA V9300) mid-dorsal vertebra in posterior view; G, *Desmatosuchus spurensis* (MNA V9300) anterior mid-caudal vertebra in posterior view. Scale bars equal 1 cm.

Figure 8: TTU P-9416, posterior dorsal vertebra of *Paratypothorax* sp. showing defined character states. A, centrum in ventral view; B, neural arch in posterolateral view showing posterior projection (pro). Scale bars equal 1 cm.

Figure 9: Scapulocoracoids and humeri of aetosaurians showing defined character states. A, *Neoaetosauroides engaeus* (PVL 3525) left scapulocoracoid in lateral view; B, *Longosuchus meadei* (TMM 31185-84a) right scapulocoracoid in lateral view; C, *Typothorax coccinarum* (UCMP 34240) distal end of left humerus in anterior view; D, *Stagonolepis olenkae* (ABIII 1175) right humerus in posterior view; E, *Aetosauroides scagliai* (PVL 2073) left humerus in anterior view. Scale bars equal 1 cm.

Figure 10: Sacra of aetosaurians showing defined character states. A, *Aetosauroides scagliai* (PVL 2073) ventral view; B, *Desmatosuchus spurensis* (MNA V9300) right lateral view; C, *Scutarx deltatylus* (PEFO 31217) ventral view. Scale bar for A equals 1 cm, for B equals 5 cm.

Figure 11: Paramedian osteoderms of aetosaurians showing defined character states. A, *Desmatosuchus smalli* (TTU P-9024) left posterior cervical osteoderm in dorsal view; B, Paratypothoracini (UCMP 139562) left cervical osteoderm in dorsal view; C, *Rioarribasuchus chamaensis* (NMMNH P-35459) left anterior caudal paramedian in dorsal view; D, *Scutarx deltatylus* (PEFO 34045) right dorsal trunk paramedian in dorsal view; E, *Lucasuchus hunti* (TMM 31100-361) right dorsal trunk osteoderm in posterior view; F, *Desmatosuchus spurensis* (MNA V9300) right dorsal trunk osteoderm in dorsal view; G, *Paratypothorax andressorum* (SMNS numbered L16) left dorsal trunk osteoderm in dorsal view; H, *Lucasuchus hunti* (TMM 31100-361) right dorsal trunk osteoderm in dorsal view; I, *Tecovasuchus chatterjeei* (TTU P-00545) right dorsal trunk osteoderm in dorsal view; J, *Stagonolepis robertsoni* (NHMUK 4789a) cast of left dorsal trunk osteoderm in dorsal view; K, *Desmatosuchus spurensis* (PEFO 26668) left dorsal trunk osteoderm in lateral view; L, *Paratypothorax* sp. (UCMP 34227) right dorsal trunk osteoderm in dorsal view. Scale bars equal 1 cm.

Figure 12: Paramedian and lateral osteoderms of aetosaurians showing defined character states. A, *Typothorax coccinarum* (PEFO 34848) left dorsal trunk paramedian osteoderm in posterior view; B, *Typothorax coccinarum* (AMNH FR 2709) left paramedian osteoderm in ventral view; C-D, *Desmatosuchus spurensis* (MNA V687) fused left anterior dorsal trunk paramedian and lateral trunk osteoderms in ventral (C) and dorsal (D) views; E-F, *Scutarx delatatylus* (PEFO 34045) right dorsal trunk paramedian osteoderm in dorsal (E) and posterior (F) views; G, *Longosuchus meadei* (TMM 31185-84B) fused right anterior dorsal trunk and lateral trunk osteoderms in posterior view. Scale bars equal 1 cm. Abbreviations: de, dorsal eminence, lo, lateral osteoderm, po, paramedian osteoderm, tt, triangular tuber.

Figure 13: Lateral osteoderms of aetosaurians showing defined character states. A, *Desmatosuchus spurensis* (MNA V9300) right dorsal trunk osteoderm in anterior view; B, *Desmatosuchus spurensis* (MNA V9300) right dorsal trunk osteoderm in dorsal view; C-D, *Aetosauroides scagliai* (PVL 2073) right dorsal trunk osteoderm in dorsolateral (C) and posterior (D) views; E, *Desmatosuchus spurensis* (MNA V9300) left anterior dorsal trunk osteoderm in posterior view; F, *Desmatosuchus spurensis* (MNA V9300) right dorsal trunk osteoderm in lateral view; G, *Redondasuchus rineharti* (MDM 20110607RRBW006#2) left dorsal trunk osteoderm in posterior view; H, *Desmatosuchus spurensis* (MNA V9300) left cervical osteoderm in lateral view; I-J, *Tecovasuchus chatterjeei* (TTU P-00545) left dorsal trunk osteoderm in dorsomedial (I) and lateral views; K, *Calyptosuchus wellesi* (UCMP 27225) left dorsal trunk osteoderm in dorsolateral view. Scale bars equal 1 cm.

Figure 14: Lateral osteoderms of aetosaurians showing defined character states. A, *Desmatosuchus smalli* (TTU P-9024) right posterior dorsal trunk osteoderm in posterior view; B, *Tecovasuchus chatterjeei* (TTU P-00545) left dorsal trunk osteoderm in posterior view; C, *Scutarx deltatylus* (UCMP 35738) right dorsal trunk osteoderm in posterior view. Scale bars equal 1 cm.
