# Supplementary material for: Revised phylogenetic analysis of the Aetosauria (Archosauria: Pseudosuchia); assessing the effects of incongruent morphological character sets
Source: PeerJ. 2016 Jan 21;4:e1583. doi: 10.7717/peerj.1583 (PMC4727975; doi:10.7717/peerj.1583)
Supplement: Supplemental Information 3 [file peerj-04-1583-s003.docx]

**APPENDIX C**

#### Tree support

Character state transformations were evaluated under both the accelerated transformation (ACCTRAN) and delayed transformation (DELTRAN) options. Synapomorphies recovered under each option are listed for each node and character states placed at the same node under both ACCTRAN and DELTRAN criteria are considered to be unambiguous synapomorphies. Underlined numbers represent characters with a C.I. of 1.000 and can be considered to be robust synapomorphies.

Unnamed node (*Revueltosaurus callenderi* + Aetosauria)

*Unambiguous synapomorphies* -- 1) lateral surface of maxilla bears a sharp longitudinal ridge (7-1); 2) ventrolateral margin of the nasal forms part of the dorsal border of the antorbital fossa (10-1); 3) postfrontal-parietal contact is restricted by a posterolateral process of the frontal (14-1); 4) anterior process of the quadratojugal forms the ventral margin of lateral temporal fenestra (16-1); 5) transverse width of frontals greater than that of the parietals at their anteroposterior mid-points (19-1); 6) basal tubera of the basicranial are clearly separated in ventral view (24-1); 7) The crown bases of the maxillary teeth are anteroposteriorly oval, but not strongly mediolaterally compressed in occlusal view (34-1); 8) articular face of the cervical centrum is round (38-1); 9) trunk vertebrae lack well-developed intervertebral articulations (hyposphene/hypantrum) (43-1); 10) anterior bar present and strongly raised om osteoderms (52-1); 11) lateral osteoderms only present in the sacral and anterior caudal regions (73-1); and 12) ventral osteoderms square and overlapping (83-2).

*Other possible synapomorphies* -- ACCTRAN: 1) shape of the maxillary tooth crown in labial/lingular view is bulbous with pointed or slightly recurved tips (35-2); 2) subglenoid ‘pillar’ absent on coracoid (46-1); and 3) acetabulum of ilium opens opens fully or mostly laterally (49-1). DELTRAN: none.

Aetosauria Marsh, 1884 *sensu* Parker, 2007. Modified by Nesbitt, 2011.

*Definition* -- The most inclusive clade containing *Aetosaurus ferratus* and *Desmatosuchus spurensis*, but not *Rutiodon carolinensis*, *Postosuchus kirkpatricki*, *Prestosuchus chiniquensis*, *Poposaurus gracilis*, *Crocodylus niloticus*, *Gracilisuchus stipanicicorum*, and *Revueltosaurus callenderi*.

*Unambiguous synapomorphies* -- 1) premaxillary teeth restricted to posterior portion of the element (3-1); 2) external nares longer than or equal to the antorbital fenestra (6-1); 3) prefrontal-parietal contact is extensive (14-2); 4) supratemporal fenestra is dorsolaterally or laterally oriented and visible in lateral view (20-1); 4) posterodorsal process of dentary more elongate than the posteroventral process (26-1); 5) anterior portion of dentary edentulous (28-1); 6) radiate patterning on paramedian osteoderms (53-1); 7) dorsal eminence of paramedian osteoderm contacts the posterior margin of osteoderm in most osteoderm rows (54-1); 8) the dorsal eminence of the anterior caudal paramedian osteoderms are low and pyramidal or rounded and knob-like (71-1); and 9) lateral osteoderms present along the entire dorsal carapace (73-2).

*Other possible synapomorphies* -- ACCTRAN: 1) dorsally projecting tuber present on articular (33-1). DELTRAN: none.

Stagonolepididae Lydekker, 1887 *sensu* Heckert & Lucas, 2000.

*Definition* -- The last common ancestor of *Desmatosuchus spurensis* and *Aetosaurus ferratus* and all of their descendants.

1) maxilla contributes to the margin of the external naris (2-1); 2) premaxilla has prominent dorsal tubercle that extends dorsally into the external naris (5-1); 3) anterior ends of the dentary prolonged into an acute rostrum (slipper-shaped) (30-1); and 4) maxillary teeth are conical in cross section (34-2).

*Other possible synapomorphies --* ACCTRAN: 1) A ventral 'chin' is present on the mandibular ramus formed by a ventral inflexion of the dentary, which covers the splenial (29-2) and 2) subglenoid pillar present on coracoid (46-1). DELTRAN: 1) dorsally projecting tuber present on articular (33-1); and 2) shape of the maxillary tooth crown in labial/lingular view is bulbous with pointed or slightly recurved tips (35-2).

Aetosaurinae Marsh 1884, *sensu* Heckert & Lucas, 2000.

*Revised Definition* – The least inclusive clade containing *Aetosaurus ferratus* but not *Desmatosuchus smalli*.

*Unambiguous synapomorphies* -- 1) transverse width of parietals greater than transverse width of the frontals (19-0); and 2), the anteroposterior diameter of the supratemporal fenestra is roughly half the size of the orbit (22-1).

*Other possible synapomorphies --* ACCTRAN: 1) pubis symphysis short, less than one-half of element length (51-1). DELTRAN: none.

Typothoracinae Huene, 1915 *sensu* Parker, 2007. Emended clade name

*Revised Definition* – The least inclusive clade containing *Typothorax coccinarum* and *Paratypothorax andressorum,* but not *Aetosaurus ferratus*, *Stagonolepis robertsoni* or *Desmatosuchus smalli.*

Note: This clade was first named Typothoracisinae (Parker, 2007); however, the formation of this name is incorrect as the root for ‘thorax’ is ‘thorac’ not ‘thoracis’ so the proper formation of this clade name is Typothoracinae. The family name Typothoracidae was first proposed by Huene (1915).

*Unambiguous synapomorphies* -- 1) width/length ratio of widest paramedian osteoderms (rows 9-11) in dorsal trunk series is greater than 3.5 (64-2); and 2) carapace is broad and discoidal in dorsal view (82-2).

Other possible synapomorphies -- ACCTRAN: 1) anterior projection of quadratojugal underlies the posterior process of the jugal and excluded from the lateral temporal fenestra (16-2); 2) articular lacks strong dorsally projecting tuber (33-0); 3) cervical vertebrae with a transversely oval articular face of the centrum (38-0); 4) transverse processes of the trunk vertebrae are elongate, more than twice as wide as the centrum (40-1); 5) neural spine height of the mid-trunk vertebrae is equal to or less than the height of the centrum (41-1); 6) proximal head of the humerus is broadly expanded transversely, with significant lateral expansion (48-1); 7) acetabulum on ilium opens fully or mostly ventrally (49-0); 8) strongly developed ventral keel on the paramedian osteoderms (56-2); 9) dorsal eminence of cervical lateral osteoderms is a moderate length, dorsoventrally flattened, slightly recurved spine (74-1); 10) mid-trunk lateral osteoderms with a strongly acute angle of flexion between the dorsal and lateral flanges (79-2); and 11) lateral flange of pelvic and anterior caudal lateral osteoderms is roughly triangular in lateral view with a semicircular ventrolateral border and a hook-like eminence (81-1). DELTRAN: none.

Unnamed node ((Paratypothoracini + (*Typothorax coccinarum* + *Redondasuchus rineharti*)).

*Unambiguous synapomorphy* -- lateral edge of the dorsal paramedian osteoderms in dorsal view are strongly sigmoidal with a strongly posteromedially oriented posterolateral corner (63-1).

*Other possible synapomorphies* – ACCTRAN: none. DELTRAN: 1) anterior projection of quadratojugal underlies the posterior process of the jugal and excluded from the lateral temporal fenestra (16-2); 2) transverse processes of the trunk vertebrae are elongate, more than twice as wide as the centrum (40-1); 3) neural spine height of the mid-trunk vertebrae is equal to or less than the height of the centrum (41-1); 4) dorsal eminence of cervical lateral osteoderms is a moderate length, dorsoventrally flattened, slightly recurved spine (74-1); 5) mid-dorsal lateral osteoderms with a strongly acute angle of flexion between the dorsal and lateral flanges (79-2); and 6) lateral flange of pelvic and anterior caudal lateral osteoderms is roughly triangular in lateral vie with a semicircular ventrolateral border and a hook-like eminence (81-1).

Unnamed node (*Typothorax coccinarum* + *Redondasuchus rineharti*)

*Unambiguous synapomorphies* -- 1) cervical vertebrae extremely shortened anteroposteriorly (37-1); and 2) surface pattern of dorsal paramedian osteoderms is reticulate (53-2).

Other possible synapomorphies -- ACCTRAN: 1) premaxilla lacks a prominent dorsal tubercle that extends dorsally into the external naris (5-0); 2) lateral surface of the maxilla is smooth, lacking longitudinal ridge (7-2); 3) lateral margin of the nasal does not form part of the dorsal border of the antorbital fossa (10-0); 4) supratemporal fenestra larger than or nearly same size as the orbit (22-0); 5) retroarticular process is longer than high (32-1); 6) ectepicondyle of the humerus proximodistally oriented foramen present on its lateral side (47-1); and 7) ratio of cervical vertebrae/paramedian osteoderms significantly less than 1:1 (58-1). DELTRAN: 1) ventral strut of paramedian osteoderms strongly developed (56-2).

Paratypothoracini Parker, 2007. Emended clade name.

*Revised Definition* -- The least inclusive clade containing *Tecovasuchus chatterjeei,* *Rioarribasuchus chamaensis,* and *Paratypothorax* *andressorum*.

Note: This clade was first named Paratypothoracisini (Parker, 2007); however, the formation of this name is incorrect as the root for ‘thorax’ is ‘thorac’ not ‘thoracis’ so the proper formation of this clade name is Paratypothoracini.

*Unambiguous synapomorphies* -- 1) caudal ribs of the caudal vertebrae attach near the base of the centrum (45-1); 2) anterior bar present, but weakly raised (52-2); 3) dorsal eminence of the paramedian osteoderms does not contact the posterior margin of the osteoderm in most rows (54-0); 5) dorsal eminence of the dorsal paramedian osteoderms is strongly offset medially (66-2); 5) dorsal flange of the dorsal lateral osteoderms is highly reduced and ‘tongue-shaped’ (76-2); and 6) dorsal eminence of the anterior and mid-dorsal lateral osteoderms is in the form of an elongate flattened horn (78-1).

Other possible synapomorphies -- ACCTRAN: 1) Transverse width of frontal wider than parietal (19-1); 2) strong posteroventral orientation of the posterior portion of the parietal forms a triangular supratemporal fenestra (21-1); 3) maxillary teeth are anteroposteriorly oval, but not strongly mediolaterally compressed in cross section (34-1); and 4) shape of the maxillary tooth crown in labial/lingular view is bulbous and partly recurved, anterior edge is concave, posterior edge straight (35-1). DELTRAN: none.

Unnamed node (SMNS 19003 + Paratypothoracini).

*Unambiguous synapomorphy* -- posterior margin of paramedian osteoderms bears a transverse, posteroventrally sloping flange (bevel) (57-1).

*Other possible synapomorphies* -- none.

Unnamed node (*Tecovasuchus chatterjeei* + *Paratypothorax*).

*Unambiguous synapomorphy* -- distinct sharp raised mediolateral ridge extends medially from dorsal eminence of paramedian osteoderm to medial osteoderm margin (65-1).

*Other possible synapomorphy* -- ACCTRAN: none. DELTRAN: ventral strut of paramedian osteoderms strongly developed (56-2).

*Paratypothorax* (*Paratypothorax andressorum* + *Paratypothorax* sp.).

*Unambiguous synapomorphies* -- 1) lateral faces of the posterior trunk vertebrae meet to form a sharp ventral edge or keel (44-1); and 2) dorsal eminence of the posterior trunk – anterior caudal paramedian osteoderms is a moderate, bulbous spike (71-2).

*Other possible synapomorphies* -- none.

Desmatosuchia Case, 1920. New clade name.

*Definition* -- The most inclusive clade containing *Stagonolepis robertsoni* and *Desmatosuchus smalli*, but not *Aetosaurus ferratus* and *Paratypothorax andressorum*.

*Unambiguous synapomorphies* -- 1) anterior portion of the premaxilla laterally expanded in dorsal view (1-1); 2) anterior portion of nasal maintains an equal width in dorsal view (9-1); 3) triangular depression on the midline suture area of the nasals absent (11-1); 4) jugal contributes to the margin of the antorbital fenestra (13-1); 5) basal tubera are nearly or completely connected (24-0); and 6) surangular bears prominent dorsal tuber (31-1).

*Other possible synapomorphies* – ACCTRAN: none. DELTRAN: 1) A 'chin' is present on the mandibular ramus formed by a ventral inflexion of the dentary, which covers the splenial (29-2); and 2) acetabulum on ilium opens fully or mostly laterally (49-1).

Stagonolepidinae Huene 1936*, sensu* Heckert & Lucas, 2000.

*Revised Definition* – The most inclusive clade containing *Stagonolepis robertsoni*, but not *Desmatosuchus spurensis* or *Paratypothorax andressorum*.

*Unambiguous synapomorphies* – 1) proximal portion of pubis bears two foramina' (50-1).

*Other possible synapomorphies --* ACCTRAN: 1) Postfrontal-parietal contact restricted by a posterolateral process of the frontal (14-1). DELTRAN: none.

Desmatosuchinae Huene 1936, *sensu* Heckert & Lucas, 2000.

*Revised Definition* – The most inclusive clade containing *Desmatosuchus smalli,* but not *Stagonolepis robertsoni*, *Aetosaurus ferratus*, or *Paratypothorax andressorum*.

*Unambiguous synapomorphies* -- 1) lateral surface of the maxilla is smooth, lacking longitudinal ridge (reversed in *Longosuchus meadei*) (7-2); 2) lateral margin of the nasal does not form part of the dorsal border of the antorbital fossa (10-0); 3) quadrate foramen entirely within quadrate bone (18-1), and 4) fewer than nine tooth positions in the dentary (27-1).

*Other possible synapomorphies* -- ACCTRAN: 1) ventral margin of the jugal strongly anterodorsally inclined in lateral view (12-1); and 2) proximal head of the humerus broadly expanded transversely, with significant lateral expansion (48-1). DELTRAN: None.

Unnamed node (*Neoaetosauroides engaeus* + Desmatosuchinae).

*Unambiguous synapomorphies* -- 1) supratemporal fenestra is roughly half the size of the orbit (22-1); 3) dorsal and ventral posteroventral processes of the dentary are roughly equal in length (26-0); and 4) anterolateral projection of the anterior bar of the dorsal paramedian osteoderms is present and elongate (reversed in Desmatosuchini) (68-1).

*Other possible synapomorphies* -- ACCTRAN: 1) ventral portion of the antorbital fossa on the maxilla is very shallow or absent (8-1); 2) retroarticular process is longer than high (32-1); and 3) ectepicondyle of the humerus proximodistally oriented foramen present on its lateral side (47-1). DELTRAN: 1) ventral margin of the jugal strongly anterodorsally inclined in lateral view (12-1).

Unnamed node (((*Adamanasuchus eisenhardtae* + *Scutarx deltatylus*) + *Calyptosuchus wellesi*)) + Desmatosuchini))).

*Unambiguous synapomorphies* -- 1) basal tubera and basipterygoid processes widely separated anteroposteriorly (reversed in *Desmatosuchus smalli* and *Scutarx deltatylus*; convergent with *Tecovasuchus chatterjeei*) (25-1); and 2) cervical vertebrae with a transversely oval articular face of the centrum (38-0).

*Other possible synapomorphies* -- ACCTRAN: 1) edentulous premaxilla (3-2); 2) edentulous premaxilla (4-2); 3) postfrontal/parietal contact absent (14-0); and 4) ratio of cervical vertebrae/paramedian osteoderms significantly less than 1:1 (58-1). DELTRAN: none.

Desmatosuchini. Case, 1920. New clade name.

*Definition* – The most inclusive clade containing *Desmatosuchus smalli,* but not *Neoaetosauroides engaeus*, *Scutarx deltatylus, Stagonolepis robertsoni*, *Aetosaurus ferratus*, *Calyptosuchus wellesi*, and *Paratypothorax andressorum*.

*Unambiguous synapomorphies* -- 1) random surface patterning of paramedian osteoderms (reversed in *Lucasuchus hunti*) (53-0); 2) in the dorsal trunk paramedian osteoderms the anterior edge of the lateral osteoderm overlaps the anterior edge of the paramedian osteoderm (62-1); 3) lacks the sharp anteromedial projection of the anterior bar (reversed in *Lucasuchus hunti*) (67-1); 4) anterior bar of the dorsal trunk paramedian osteoderms lacks scalloping of the anterior margin on the medial side of the osteoderm (69-1); 5) dorsal eminence of the cervical lateral osteoderms is in the form of a moderately long, faceted, slightly recurved spine (74-2); 6) rectangular dorsal flange of the dorsal lateral osteoderms (76-0); 7) approximately 90 degree angle between the dorsal and lateral flanges of the mid-trunk lateral osteoderms (79-1); 8) dorsal trunk lateral osteoderms strongly asymmetrical with the dorsal flange longest (80-1); and 9) overall shape in of the dorsal carapace in dorsal view is moderately spinose (82-1).

*Other possible synapomorphies*- ACCTRAN: 1) post-temporal fenestra is absent (23-1); 2) ventral ‘chin’ of the mandibular ramus present and formed by a ventral inflexion of the splenial (29-1); 3) in the paramedian osteoderms dorsal to the cervical and anterior trunk vertebrae, lateral edge articulation with lateral osteoderms is dorsoventrally thickened, angled contact, with deeply incised interdigitation (='tongue and groove') (60-1); 4) dorsal eminence shape in the cervical paramedian osteoderms are a low pyramidal or rounded boss, or elongate keel (61-1); 5) the anterior bar of the trunk distal paramedian osteoderms lacks an anterolateral pro69-jection (68-2); 6) dorsal eminence in the mid-trunk osteoderms is a conical spike (78-2); 7) lateral flange of the pelvic and anterior caudal lateral osteoderms are rectangular and ventral to a well-developed spine (81-2); and 8) ventral osteoderms absent (83-0). DELTRAN: ratio of cervical vertebrae/paramedian osteoderms significantly less than 1:1 (58-1).

Unnamed node (*Longosuchus meadei* + Desmatosuchini).

*Unambiguous synapomorphies* -- 1) cervical paramedian osteoderms are longer than wide (57-1); and 2) adjacent paramedian and lateral cervical osteoderms are often fused (59-1).

*Other possible synapomorphies* – ACCTRAN: none. DELTRAN: 1) ventral portion of the antorbital fossa on the maxilla is very shallow or absent (8-1); 2) post-temporal fenestra is absent (23-1); 3) ventral ‘chin’ of the mandibular ramus present and formed by a ventral inflexion of the splenial (29-1); 4) proximal head of humerus broadly expanded transversely with a significant lateral expansion (48-1); 5) in the paramedian osteoderms dorsal to the cervical and anterior trunk vertebrae, lateral edge articulation with lateral osteoderms is dorsoventrally thickened, angled contact, with deeply incised interdigitation (='tongue and groove') (60-1); 6) dorsal eminence shape in the cervical paramedian osteoderms are a low pyramidal or rounded boss, or elongate keel (61-1); 7) the anterior bar of the trunk dirsal paramedian osteoderms lacks an anterolateral projection (68-2); 8) dorsal eminence in the mid-dorsal osteoderms is a conical spike (78-2); 9) lateral flange of the pelvic and anterior caudal lateral osteoderms are rectangular and ventral to a well-developed spine (81-2); and 10) ventral osteoderms absent (83-0).

Unnamed node (*Sierritasuchus macalpini* + Desmatosuchini).

*Unambiguous synapomorphies* -- 1) neural spine height of the mid-dorsal vertebrae is low, equal to or less than the height of the centrum (41-1) and 2) dorsal eminence of dorsal paramedian osteoderms is centralized (66-0).

*Other possible synapomorphies* -- ACCTRAN: 1) quadrate foramen positioned between the quadrate and quadratojugal (18-0); 2) supratemporal fenestra larger than or nearly same size as the orbit (22-0); 3) lower posteroventral process of the dentary is longer than the upper process (26-2); 4) dorsal tuber of surangular is absent (31-0); 5) articular lacks strong dorsally projecting tuber (33-0); and 6) hyposphene/hypantrum present in dorsal vertebrae (43-0). DELTRAN: none.

Unnamed node (*Lucasuchus hunti* + *Desmatosuchus*).

*Unambiguous synapomorphies* -- 1) dorsal eminence of the paramedian osteoderms almost never contacts the posterior osteoderm margin (54-0); and 2) posterior face of the dorsal trunk lateral osteoderms lack a ventral emargination (77-0).

*Other possible synapomorphy* -- ACCTRAN: 1) articular face of the cervical vertebral centrum is subrectangular (38-2). DELTRAN: none.

*Desmatosuchus* Case 1920 (= *Desmatosuchus smalli* + *Desmatosuchus spurensis*).

*Unambiguous synapomorphies* -- 1) osteoderms possess a depressed anterior lamina rather than a raised anterior bar (52-3); 2) dorsal eminence of the cervical lateral osteoderms is a greatly elongated horn (74-3); and 3) anteriormost dorsal trunk lateral osteoderms bear a mound-like dorsal eminence (75-1).

*Other possible synapomorphies* – ACCTRAN: none. DELTRAN: 1) postfrontal-parietal contact absent (14-0); 2) retroarticular process is longer than high (32-1); 3) articular lacks strong dorsally projecting tuber (33-0); 4) articular face of the cervical vertebral centrum is subrectangular (38-2); and 5) hyposphene/hypantrum present in trunk vertebrae (43-0).

Unnamed node (*Calyptosuchus wellesi* + (*Adamanasuchus eisenhardtae* + *Scutarx deltatylus*)).

*Unambiguous synapomorphies* -- 1) acetabulum on ilium opens fully or mostly ventrally (49-0); 2) ventral strut of the paramedian osteoderms weakly developed (56-1); and 3) width/length ratio of widest paramedian osteoderms is between 3.01 and 3.5 (64-1).

*Other possible synapomorphies* -- ACCTRAN: 1) dentary tooth count of nine or more (27-0); and 2) two pubic foramina (50-1). DELTRAN: none.

Unnamed node (*Adamanasuchus eisenhardtae* + *Scutarx deltatylus*).

*Unambiguous synapomorphies* -- 1) anterolateral projection of the anterior bar of the dorsal paramedian osteoderms is present and elongate (70-1).

*Other possible synapomorphies* -- ACCTRAN: none. DELTRAN: none.

## REFERENCES

Case EC. 1920. Preliminary description of a new suborder of phytosaurian reptiles with a description of a new species of *Phytosaurus*. *Journal of Geology* 28:524–535.

Heckert AB, Lucas SG. 2000. Taxonomy, phylogeny, biostratigraphy, biochronology, paleobiogeography, and evolution of the Late Triassic Aetosauria (Archosauria: Crurotarsi). *Zentralblatt für Geologie und Paläontologie Teil I* 1998 Heft 11–12:1539-1587.

Huene Fv. 1915. On reptiles of the New Mexican Trias in the Cope collection. *American Museum of Natural History Bulletin* 34:485-507.

Huene Fv. 1936. The constitution of the Thecodontia. *American Journal of Science* 32:207-217.

Lydekker R. 1887. The fossil Vertebrata of India. *Records of the Geological Survey of India* 20:51–80.

Marsh OC. 1884. The classification and affinities of dinosaurian reptiles. *Nature* 31:68–69.

Nesbitt SJ. 2011. The early evolution of archosaurs: relationships and the origin of major clades. *Bulletin of the American Museum of Natural History* 352:1–292.

Parker WG. 2007. Reassessment of the aetosaur “*Desmatosuchus*” *chamaensis* with a reanalysis of the phylogeny of the Aetosauria (Archosauria: Pseudosuchia). *Journal of Systematic Palaeontology* 5:1–28.
